# The untold stories of the speech gene, the *FOXP2* cancer gene

**DOI:** 10.18632/genesandcancer.169

**Published:** 2018-01

**Authors:** Maria Jesus Herrero, Yorick Gitton

**Affiliations:** ^1^ Center for Neuroscience Research, Children's National Medical Center, NW, Washington, DC, USA; ^2^ Sorbonne University, INSERM, CNRS, Vision Institute Research Center, Paris, France

**Keywords:** FOXP2 factor, oncogene, cancer, prognosis, language

## Abstract

*FOXP2* encodes a transcription factor involved in speech and language acquisition. Growing evidence now suggests that dysregulated *FOXP2* activity may also be instrumental in human oncogenesis, along the lines of other cardinal developmental transcription factors such as *DLX5* and *DLX6* [1–4].

Several *FOXP* familymembers are directly involved during cancer initiation, maintenance and progression in the adult [5–8]. This may comprise either a pro-oncogenic activity or a deficient tumor-suppressor role, depending upon cell types and associated signaling pathways. While *FOXP2* is expressed in numerous cell types, its expression has been found to be down-regulated in breast cancer [9], hepatocellular carcinoma [8] and gastric cancer biopsies [10]. Conversely, overexpressed *FOXP2* has been reported in multiple myelomas, MGUS (Monoclonal Gammopathy of Undetermined Significance), several subtypes of lymphomas [5,11], as well as in neuroblastomas [12] and ERG fusion-negative prostate cancers [13]. According to functional evidences reported in breast cancer [9] and survey of recent transcriptomic and proteomic analyses of different tumor biopsies, we postulate that *FOXP2* dysregulation may play a main role throughout cancer initiation and progression. In some cancer conditions, *FOXP2* levels are now considered as a critical diagnostic marker of neoplastic cells, and in many situations, they even bear strong prognostic value [5]. Whether *FOXP2* may further become a therapeutic target is an actively explored lead. Knowledge reviewed here may help improve our understanding of *FOXP2* roles during oncogenesis and provide cues for diagnostic, prognostic and therapeutic analyses.

## INTRODUCTION

*FOXP2* belongs to the extensive family of more than forty-three Forkhead box-winged helix transcription factors organized into nineteen sub-families. They are endowed with both activating and, more often, repressing transcriptional activities [14]. Conserved *FOXP2* expression is detected throughout several developing tissues, including the brain [15] [16] [17]. This organ has been a major focus of *FOXP2* research as a gene involved in language and speech acquisition in modern humans, vocalizations in mouse and other communicative skills [18].

Clinically, chromosomal lesions involving the *FOXP2* locus are associated with impaired brain development and neuronal differentiation, and give origin to complex neural disorders - the most salient impacting language processing and speech [19]. Genetic invalidation of the murine *Foxp2* leads to severe developmental delays, motor defects, absence of ultrasonic sounds that juveniles emit when separated from their mothers, and premature death [20]. However, *FOXP2* is also expressed in a large spectrum of other embryonic, postnatal and adult tissues, where its dysregulation has been observed to be associated with cancer conditions. The present manuscript reviews the features of *FOXP2* genomic context, its transcripts, its protein isoforms and targets, and data that substantiate the notion of a critical contribution of *FOXP2* to cellular barriers against cancer progression. Such information provides an integrated view of available evidence which may allow to append “oncogene” to its “language” gene designation.

### 1 Structural characterization of the transcription factor-encoding *FOXP2* gene

#### 1.1 DNA

##### 1.1.1 Genomic landscape of *FOXP2*: a tumorigenic hotspot?

**Figure 1:**
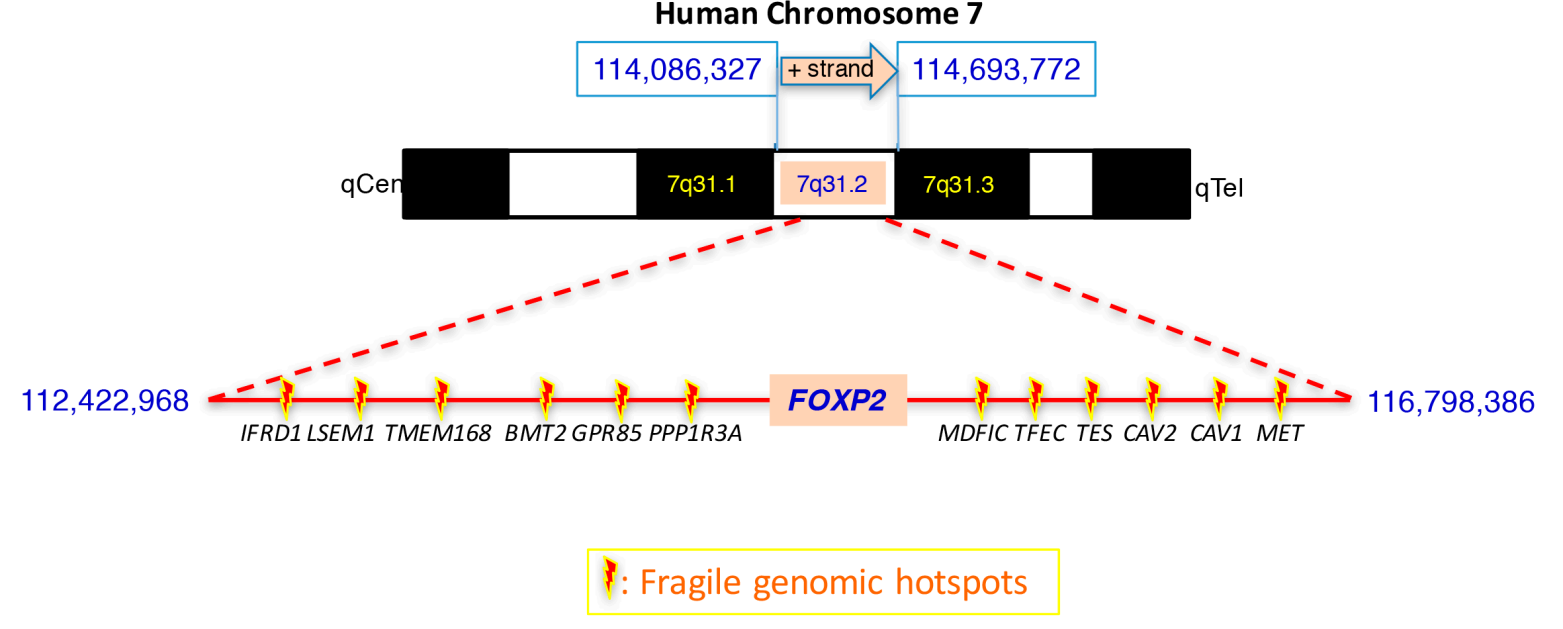
DNA HUMAN *FOXP2* LOCUS (NCBI ID: 93986; Atlas ID: 40633). Location of the *FOXP2* locus on the long arm of chromosome 7 band 7q31.1 (fragment, ENSEMBL coordinates GRCh38:CM000669.2). *FOXP2* is located within a 5 Mb-large region of fragile genomic hotspots involving the highlighted neighboring genes.

*FOXP2* has been designated previously as follows: *SPCH1* (“speech and language disorder 1”), *TNRC10* (“trinucleotide repeat containing 10”), *CAGH44* (“CAG repeat protein 44”), and *DKFZp686H1726* (HNGC nomenclature). The genomic context of human *FOXP2* is the large arm of the chromosome 7, on the forward strand, from Ensembl coordinates chr7:114,086,327 to 114,693,772 in GRCh38:CM000669.2 (June 2017; Ensembl) (Figure 1). Previously UCSC had located it in chr7:114,414,997-114,693,768 in GRCh38/hg38 (Dec. 2013; UCSC).

This locus abuts the junction of Giemsa-negative and positive bands q31 and q32. Noticeably, these bands are prone to somatic chromosomal instability and subsequent rearrangements, favoring tumorigenesis and cancer progression [21,22]. Furthermore this region has been reported to harbor fragile genomic hotspots impacting neighboring genes [23], as depicted in the bottom part of Figure 1. In the vicinity of the *FOXP2* locus, within 5Mb, neighboring protein-encoding genes include *PPP1R3A, GPR85, BMT2, TMEM168, LSEM1, IFRD1* centromerically; and *MDFIC, TFEC, TES, CAV2, CAV1* and *MET,* telomerically. Among these genes, *TES/TESTIN* and *MET* are of particular oncogenic interest through their role as a 7q31 hotspots reported for genomic instability leading to invalidation of these tumor-suppressor (*TES*) and pro-oncogenic (*MET*) factors [24,25].

Three farther hotspot candidate genes have been involved in a variety of pathological conditions when deficient, including neoplasia:

i) The *SMO* (SMOOTHENED gene), at 7q32, a frizzled-class receptor belonging to the SONIC HEDGEHOG (SHH) pathway, associated with oncogenic conditions including basal cell carcinoma, malignant glioma, medulloblastoma, leukemia, and cancers of the breast, lung, pancreas, and prostate [26].

ii) the *B-RAF* proto-oncogene at 7q34, a serine/threonine kinase associated with leukemia, melanoma, thyroid, ovarian, colon and lung cancer [27].

iii) the *EZH2* (*ENHANCER OF ZESTE 2*) polycomb repressive complex 2 subunit, at 7q36, a transcriptional co-repressor, frequently overexpressed in many cancer types, including prostate, breast, bladder, ovarian, lung, liver, brain, kidney, gastric, oesophageal, and pancreatic cancers and melanoma [28].

Whether these oncogenic situations result from or depend upon isolated or combined defects of *FOXP2* and its neighbors remains unclear. We have collated observations from cancer databases on genomic lesions involving genes in the vicinity of *FOXP2* in section 5.2 (see further).

##### 1.1.2 Epigenomic landscape of *FOXP2*

###### 1.1.2.1 Contradictory evidence for imprinting of the *FOXP2* locus

Dysregulation of parental allele-specific expression of genes, resulting from altered imprinting marks deposited onto specific loci, has often been associated with altered copy number variation and early stages of oncogenic processes. Loss of imprinting in both human and mice, impacting either global genomic territories or more specific loci, is a precious diagnostic hallmark for the earliest events of malign transformation - and the most frequent defect during tumorigenesis. To assess the susceptibility of the *FOXP2* locus to genomic lesions which may have deleterious oncogenic consequences, we first reviewed the status of the imprinting of this locus.

The *FOXP2* locus is subject to conflicting interpretations with regards to the imprinting status. A first series of parental origin of polymorphisms expressed in a cohort of patients has uncovered a bias towards a maternal imprinting leading to an exclusive paternal expression of *FOXP2* [29]. However, other authors have revisited this issue using more sensitive methods consisting in decoding the parental origin of the *FOXP2* transcripts [30]. Their observations were consistent with a biallelic expression of *FOXP2*, ruling out any role for imprinting. So far, direct bisulfite sequencing, which would bring more sensitive evidence for differential imprints on the *FOXP2* locus, has not been reported - although efforts to gain such insight are appearing as in the MethCNA database [31]. In contrast, a neighboring region on 7q31 (*GRM8*) is imprinted [32]. Alternatively, an indirect imprinting mechanism was uncovered which influences the parental allelic expression of *FOXP2* in the immune cell lineage of one individual afflicted by verbal dyspraxia [33]. A regulatory region as far as 3Mb upstream of *FOXP2* harbors a putative regulatory element that controls *FOXP2* expression level and is subjected to parental imprinting. Whether this mosaic process concerns neurons involved in language remains to be determined. Along the same line of analysis, the rare case reports involving loss of heterozygosity of *FOXP2* have so far been associated with neuro-developmental disorders but not with cancer [34].

###### 1.1.2.2 The *FOXP2* locus, a target cluster for cancer-associated epigenomic conversions

In cancer, loss of expression of genes occurs frequently by hypermethylation of promoter *CpG* islands. Transcriptomic analysis of twelve genes within the 4.12Mb region centered around *FOXP2* (Figure 1) in primary prostate cancer cells has uncovered a common trend towards severe down-regulation of this set of genes [35]. This phenotype is associated with H3K27me3 hyper-methylation of *CpG* islands in promoters of this domain, with a concomitant de-acetylation, generalized to the whole domain. Clinical samples displayed identical cancer-associated epigenetic changes of these clustered genes. *FOXP2* thus belongs to a domain featuring cancer epigenome consolidation, priming clustered genes for generalized dysregulation through alteration of chromatin accessibility to transcription. The mechanism linking this transformation to a growth advantage in pre-oncogenic cells remains to be elucidated. Consistently, some of the genes in this cluster have roles in neoplasia, including *MET* (see above) whose hypomethylation and acetylation have been associated with its high expression in some cancers [24]; as well as *FOXP2* (detailed below in section 5).

##### 1.1.3 Cis-regulatory control of *FOXP2*: a target for cancer signaling cascades?

###### 1.1.3.1 *FOXP2* Promoters

Somatic epigenetic lesions contribute to major disruptive processes involved in the transition from pre-oncogenic to oncogenic conditions. Some of these events are of critical importance in normal cellular physiology as hallmarks for regulation of the transcription of associated loci. Both upstream and downstream regulatory elements have been identified as impacting *FOXP2* expression [36].

**Figure 2:**
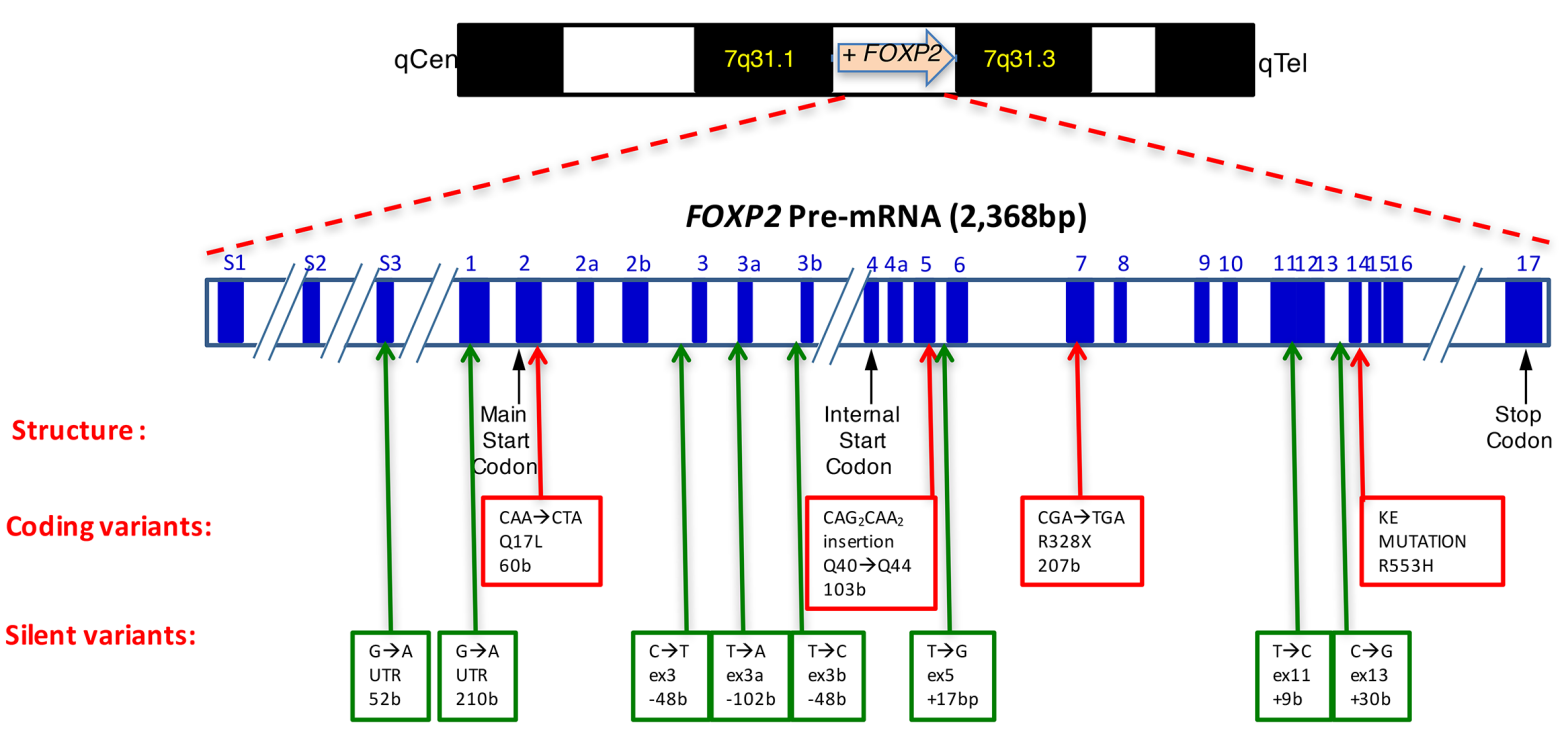
RNA HUMAN *FOXP2* pre-mRNA structure. It encodes for 17 exons (blue segments), with exon 1 being the first in the predominant *FOXP2* isoform. Bottom part: location of mutational variants assembled from different sources [19,131,176,177], including DECIPHER database.

Combining data mined from Ensembl and Genomatix (version 3.9), we have identified thirteen alternative promoters which have been experimentally validated as controlling the transcription of the twenty-seven isoforms from the *FOXP2* locus:

• Seven of these promoters are centromeric to the transcription start site of the longest and predominant product (*FOXP2-201*, ENST00000350908.8, NM_014491; Figure 2). This product was previously designated as *FOXP2_001.*

• Two promoters telomeric to the most downstream transcript were identified and functionally validated [37].

• Three promoters are intronic (Introns 1, 4, 13),

• One promoter is exonic (Exon 1).

In human tissues four alternative promoters have been validated *in vitro* - those within exons S1 and exon 2 (Figure 2) being responsible for ubiquitous and basal distribution of *FOXP2* transcripts [38]. It remains to be deciphered why three of these promoters engage *FOXP2* production from the same transcriptional start site.

The complexity of this configuration suggests multiple levels of control of *FOXP2*, which may be relevant to oncogenic processes. In the next paragraph, we focus upon the promoter which controls the most abundant form of *FOXP2* transcripts, emphasizing oncogenic signaling. This does not rule out an oncogenic role for the other promoters and associated RNA isoforms.

###### 1.1.3.2 Regulatory elements associated with *FOXP2* expression

Centromeric regulatory elements place *FOXP2* under cancer-prone signaling cascades. Using Genomatix, we screened for the strongest consensus binding sites of different transcription factors mediating oncogenic signaling. We researched only one (Genomatix ID# GXP_94278, position 7:114,413,997-114,415,423; 1427bp) of the thirteen experimentally validated promoters as conditioning the production of the predominant transcript (*FOXP2*_201) found in most cell types. We refer the reader to these types of analytic tools to examine whether other, less expressed, transcripts might fall under the control of the alternative promoters. This GXP_94278 promoter has been experimentally validated and harbors candidate binding sites for effector transcription factors of diverse oncogenic signaling pathways, from which we selected TP53 and LEF-1:

• TP53: *TP53* is a ubiquitous tumor-suppressor gene reported to be mutated in half of human cancers. The encoded factor P53 exerts its protective roles through a series of effectors, which broadly prevent excessive proliferation by dampening upon cell cycle progression and by inducing growth arrest in over-proliferating cells undergoing a neoplastic transformation by triggering apoptosis. The main activating event of P53 is DNA damage associated with oncogenic initiation. We found a bona fide binding site for P53 spanning promoter GXP_94278, positions 681-705 with a taagCAAAcccaagacaagcatttc sequence (with the core in capitals). Another position lies at chr7: 114,060,411-114,060,421 - (TAGGCAGGTCT), which is identified by the QIAGEN promoter analysis tool as a P53 binding site in human cells, among 200 other transcription factors susceptible to bind the upstream territory of the *FOXP2* locus. These elements are compatible with the notion that *FOXP2* expression status may be a direct target of TP53 activity.

• LEF1 (Lymphoid Enhancer Binding Factor 1): We emphasized our analysis on this factor because it is the downstream effector of WNT signaling in numerous normal and pathological conditions - including during oncogenesis. In the *Foxp2* genomic region at least six Lef1 binding sites were common between zebrafish, mouse and humans [39]. One study in zebrafish found a promoter controlling *Foxp2* expression and detected a matching orthologous sequence in the human genome [40]. They identified the sequence (zebrafish: ttgtgggctGCTTTCATCtgtgggttaa; orthologous to human: atgatcagtGCTTTCATCtttattttaa) at −8.5kbp upstream from human *FOXP2_201* transcription start site. BLASTing this sequence with a more recent genome version locates its exact 17bp match within the first *FOXP2* intron, at position 7: 114,417,683-114,417,710. Furthermore, the BLAST returns a perfect match on another chromosome at position 12: 90,502,826-90,502,988 - but without any obvious gene encoding sequence. The possibility remains open that it acts as a long-range cis-regulator. To further our understanding of a putative LEF1 regulation of *FOXP2* expression we screened the upstream GXP_94278 promoter (see above). We found a set of twelve binding sites with high scores, including a stretch of two on the plus strand, separated by 14bp - in which a minus strand site is also located. While speculative, this configuration opens the possibility for a putative LEF1-dependent regulation of *FOXP2* transcription associated with human oncogenesis. Such regulation could be independent or initiated by WNT signaling [41], in a cascade which may involve a WNT→ LEF1→ *FOXP2* activation sequence.

Further analyzing the possibility of oncogenic factor binding to *FOXP2* regulatory elements may yield new cues relevant to oncogenesis. We refer the reader to recent studies describing FOXP2 involvement during oncogenic processes for other major oncogenes, including *MYC* [9].

A recent genomic analysis reports the presence of two additional regulatory elements with enhancer function in the telomeric territory separating *FOXP2* from its neighbor, *MDF1C* [37]. These enhancers have been observed to be disrupted in a child with language and speech disorder. The requirement for these two elements in driving proper *FOXP2* expression levels was functionally validated in human cell lines [37]. These data lend support to the hypothesis that *FOXP2* expression falls under a large array of regulatory elements, which may increase the probability of dysregulation during oncogenic processes.

###### 1.1.3.3 A *FOXP2* intragenic regulatory element: an oncogenic target?

The *FOXP2* locus hosts many sequences which act as hallmarks of insulating regions [42]. Browsing the regulatory build channel of ENSEMBL for human *FOXP2* shows that at least ten CTCF regulatory binding sites are distributed across numerous introns, and also outside of the locus. These ‘CTCF’ regions are expected to insulate *FOXP2* intragenic regulatory elements from acting over long distance loci [43]. Hence, regulatory elements located within *FOXP2* would be expected to locally impact *FOXP2* transcription. In *H. sapiens FOXP2* intron 8, a single nucleotide substitution at position Hsa7:114,076,877 was identified, which was not present in the *H. neanderthalensis* genome [44]. The modern allele favored the binding of the neuronal-specific transcription factor POU3F2/OCT3, which promoted *FOXP2* transcription - in contrast to the ancestral allele, where POU3F2/OCT3 binding was inefficient. The authors characterized this site as a putative internal regulatory element dedicated to enhancing neuronal expression of *FOXP2* under the control of POU3F2/OCT3. While the role of this evolutionary modification in language acquisition remains to be completely elucidated, the fact that it involves POU3F2/OCT3 activity bears some relevance with brain oncogenesis. Indeed, POU3F2/OCT3 overexpression has been correlated with neuroblastoma and glioblastoma in both human brain and neuroblastoma-derived cell lines (SH-SY5Y) [12,45,46].

##### 1.2 RNA

###### 1.2.1 Transcripts

####### 1.2.1.1 Description

Transcription from *FOXP2* yields a 2,368bp long pre-messenger RNA (from 114,415,055 to 114,690,100) (Figure 2). It harbors twenty-seven splice variants - four transcripts being untranslated. The nineteen coding ones range from 625b to 8300b due to alternative splicing sites throughout the precursor transcript. The prevalent human isoform encodes for a 715AA protein from 17 exons (Isoform *FOXP2-201*, ENST00000350908.8, CCDS 43635.1, NM_014491, Uniprot O15409), which have been detailed elsewhere [38]. Furthermore, four antisense non-coding transcripts have been characterized. The composition of seventeen transcript variants is detailed and updated on a dedicated NCBI page, and available from the human FOXP2 Ensembl page. Genomic comparisons have shown that *FOXP2* is the gene harboring the most ultraconserved sequences in its introns, suggesting a wide array of putative regulatory elements. *FOXP2* has fusion genes with *COG5* (7q22.3), *RCF3 (13q13.3), RPL36* (19p13.3) and *SFTPB* (2p11.2).

####### 1.2.1.2 Exception

*FOXP2* sequence analysis of one familial mutation (R553H) [19] has led to the discovery of a longer transcript endowing FOXP2 with an unusual poly-glutamine/poly-proline stretch, forty CAG/CCG repeats long, that has been found to be aggregated when ectopically expressed in COS cells [47].

One of the functional consequences of these expansions is the initiation of a cellular stress signaling cascade. Mechanistically, FOXP2 is a binding partner for the nuclear translocation of POT1 (Protection of Telomeres I). FOXP2 promotes the nuclear translocation of POT1, but the mutated FOXP2(R553H) protein related to speech-language disorder, partially prevents it [48,49]. This may account for the altered distribution and function of this FOXP2 mutant form which cannot exert its nuclear functions.

###### 1.2.2 Post-transcriptional regulation of FOXP2 mRNA

*FOXP2* mRNAs are numerous and subjected to intensive splicing. While this property may in itself constitute a mode of regulation of *FOXP2* expression we have focused here on microRNA-mediated regulation of *FOXP2*. We have surveyed both predicted and validated miRs and identified a set of *FOXP2*-targeting microRNAs from the following databases: targetscan 7.1, mirBase 21, mirdb. Close to 284 miRs are predicted to target human *FOXP2*, including 44 with a score above 95%.

Of particular clinical interest is miR-3666 (NR_037439), a mirtron located within the *FOXP2* locus at 7 [+]:114653345-114653455 (GRCh 38) in intron 9. This intronic transcript is spliced out from all *FOXP2* pre-mRNAs isoforms. While not confidently annotated, the Targetscan database proposes its analysis with regards to a significantly close family of miRs: miR-130-3p/301-3p/454-3p. Such a co-expression scheme raises several issues. First, both miR-3666 and *FOXP2* play a larger regulatory role than expected by examining the sole impact of *FOXP2* on transcription: putatively downregulated targets of miR-3666 may complexify the impact of *FOXP2*. In particular, repressive role of *FOXP2* on target genes may be due in part to miR-3666 and begs for a thorough analysis of the direct binding of FOXP2. Next, alternative start sites of transcription combined with alternative splicing might bring an unexpected level of complexity in the balance between the host gene and its encoded microRNA. Lastly, mutations in the *FOXP2* locus might be consequential for the phenotype whether each or only one of these elements are concerned. Recently, computational evidence has been proposed for an auto-repression of *FOXP2* by miR-3666 in cell lines, and uncovered a set of several hundred genes conjointly targeted by both, with a bias toward genes putatively involved in schizophrenia and/or autism spectrum disorder [50]. In the context of cancer progression, miR-3666 has been associated with several cancer types and displayed reduced transcripts levels in lung [51], thyroid [52] and pituitary cancer cells [53]. However, how defective *FOXP2* expression associated with miR-3666 misexpression might promote oncogenic initiation, maintenance, or aggressiveness remains to be assessed.

Additionally, other microRNAs might be associated with dysregulation of *FOXP2* expression levels in oncogenic conditions. One frequent candidate is the miR-190. Of its four isoforms (a, a-3p, a-5p, b), miR-190a has been experimentally validated and located on the plus strand of chromosome 15, within the second intron of the gene *TALIN2* (*TLN2)*. The salient feature of this host gene is that it has been demonstrated to be down-regulated in cancer cell lines and biopsies. We discuss the functional consequences of this observation in sections ‘entity’ devoted to the hepatocellular carcinoma [54], breast cancer [55] and gastric cancer [10].

Conversely, up-regulated miR-190 has been reported in a variety of conditions such as bladder cancer, breast cancer, lung cancer, liver cancer, and colorectal cancer, as well as in bronchial epithelial cell cancerization induced by arsenic [56-61]. Noticeably, in glioblastomas and osteosarcomas in *in vivo* models of human cancer in immune-compromised mice, up-regulation of miR-190 led to prolonged tumor dormancy [55]. Altogether these observations in diverse oncogenic conditions point to the susceptibility of *FOXP2* expression levels to the activity of miR-190, which in some cases has been associated with oncogenic initiation.

In another large scale oncogenic study [9], *FOXP2* expression level has been shown to directly depend upon the activity of a coordinated set of microRNAs. The authors observed that the malignancy of breast cancer cells (BCCs) was enhanced upon exposure to incoming mesenchymal stem cells populating the breast tumor stroma. This interaction triggers the activation of a TWIST-dependent signaling cascade, which has two mechanistic consequences (Figure 6). First, it activates a set of adhesion-related genes in BCCs. Second, TWIST activates two waves of miRs: the 199a-214 cluster and a set of four other microRNAs (miR-762, miR-1915, let-7b, and miR-34a). All these miRs share *FOXP2* as a validated target, leading to the down-regulation of this gene. Subsequently, this cascade converged on and repressed the expression of *FOXP2* promoting cancer stem cell (CSC) and metastatic traits. This condition correlated with poor survival in breast cancer.

##### 1.3 Protein

###### 1.3.1 Biochemical properties

**Figure 3:**
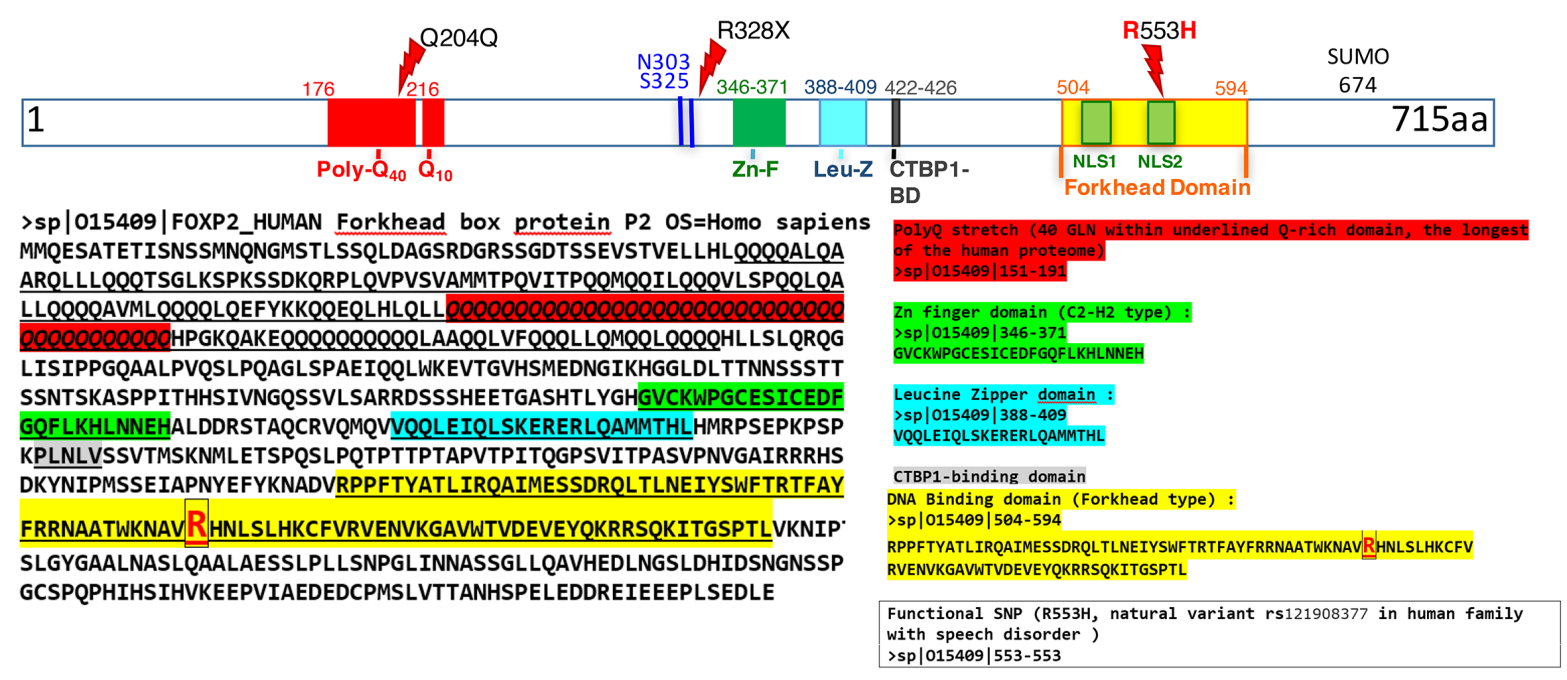
PROTEIN Human FOXP2 main protein isoform (Uniprot O15409). Structural and functional domains are highlighted. The Forkhead P2 domain harbors two nuclear localization signals (‘NLS’) [70]. Two major mutated variants are indicated above, with R328X interrupting the protein and R553H altering its subcellular localization (KE family verbal dyspraxia mutation). The two human lineage-specific aminoacids N303 and S325 are indicated in blue. The sumoylation site (K674) is indicated. A Q204Q substitution observed in multiple cancers is discussed in Figure 5.

A 715-aa transcription factor (Uniprot #O15409) is encoded by the main *FOXP2* mRNA isoform *FOXP2_201* (Figure 2). It features five main functional domains illustrated by Figure 3. The molecular mass nears 80kDa and its DNA binding activity requires dimerization. The 100-aa Forkhead domain (or ‘winged-helix’) is located in the C-terminal part and accounts for FOXP2 DNA binding activity, as has been detailed upon determination of the crystal structure of its interaction with DNA [62]. A detailed comparison of the protein structures across families of Forkhead factors has been reviewed elsewhere [63]. Biostructural analysis has identified a *FOXP2* consensus binding sequence as 5’-CAAATT-3’ [62]. Furthermore, swapping events involving the N-terminal domain adapt FOXP2 interactions with other monomers, including with its paralogs FOXP1 and FOXP4, which may provide functional plasticity to FOXP2 activity depending upon the cellular context [64]. A recent structural dissection of FOXP2 DNA binding has identified three rate and affinity modalities, respectively enabling fast genome browsing, medium target site detection and strong binding to best affinity sites engaging *FOXP2* into transcriptional activity [65]. The zinc finger and leucine zipper domains have been hypothesized to be involved in these interactions [65,66].

Furthermore, FOXP2 harbors a binding domain for the co-repressor CtBP1 (C-Terminal Binding Protein-1, Figure 3) which has been experimentally validated [34,66] and may be involved as a tumor suppressor in oncogenic processes through interaction with the BRCA1/2 breast cancer oncofactors [67,68].

FOXP2 may also behave as a chromatin bookmarking agent since it has been reported to associate with the NuRD chromatin remodelling complex which, furthermore, harbors HDAC components responsible for the repressing activity of FOXP2 [69].

Another structural feature of FOXP2 is the presence of two nuclear localization signals (NLS) embedded within the Forkhead domain. These may force FOXP2 translocation from the cytoplasm to the nucleus as demonstrated by cellular mutagenesis assays [70]. Interestingly, the R553H mutation of the KE family (see Figures 2 and 3, and pathology section 4 below) has been demonstrated to hinder FOXP2 nuclear localization due to its invalidation of the C-terminal NLS [70].

Finally, the N-terminus of FOXP2 displays the longest poly-glutamine tract of all known proteins - a distinct feature of protein misfolding disorders impacting the nervous system [34].

The FOXP2 protein undergoes a major post-translational modification by SUMOylation (Small Ubiquitin-like Modifier). It has been demonstrated to be a critical regulatory mechanism of FOXP2 activity [71]. Interaction between FOXP2 and PIAS3, a critical component of this modification, results in the addition of a SUMO group to FOXP2 at the highly conserved position K674 (Figure 3) by PIAS3 [72]. This leads to the massive redistribution of FOXP2 into nuclear speckles of granular heterochromatin [34]. However, the functional importance of this modification remains to be clarified since *PIAS3* invalidation does not prevent FOXP2 transcriptional repressive activity in some cells (Hela and HEK293), [71] but does impact it in other cells (MCF7 and SH-SY5Y, but also HEK293) [72]. Noticeably, the SUMOylation of the murine Foxp2 had been previously hypothesized to promote the dissociation of the Foxp2 dimer from its target promoter, leading to an overall reduction of Foxp2 transcription suppressive activity [74].

###### 1.3.2 Functional features

FOXP2 has been demonstrated to exert both promoting and, more often, repressing activities of the transcription of target genes. This suppressive activity might be accounted for by the Zinc finger domain [69]. Combination of chromatin immunoprecipitation with microarray analysis in cells dissected from fetal human brains has identified a strong consensus for FOXP2 response element in target loci [75]. Interestingly, it has been observed that the human ortholog displays a unique substitution at position 303 of this transcriptional repressor domain (Thr→ Asn), which sets it apart from all other species [76]. Whether this modification bears functional relevance remains to be determined.

A survey of directly bound loci yielded candidate FOXP2-binding sequences with a core underlined within CAAATT as the most probable target, even if some other alternative sites were also reported [62,75,77,78]. Collectively, most studies unveil a predominantly repressor role for FOXP2 upon transcription of target genes, even if some exceptions report activating properties [17,75,78-82].

One major functional aspect of FOXP2 activity stems from its dimerization with its paralogs FOXP1 and FOXP4. Indeed, different combinations of FOXP1/2/4 dimerization severely affect gene expression [64]. This property may have oncogenic consequences. FOXP1 and FOXP2 have been observed to be widely co-expressed in specific territories [83] and to co-operate at least during development [84]. Deficiency of both factors has been involved in malformative processes leading to autistic, language and cognitive deficiencies, among others [85]. Additionally, dysregulated FOXP1 expression has been associated to deleterious oncogenic activities, including hepatocellular carcinoma, breast, renal, prostate and endometrial cancer [86-93]. Similarly, FOXP4 has been shown to interact with FOXP2 in several developmental processes, such as neuronal differentiation and migration [94]. *FOXP4* expression was aberrant in several breast cancer cell lines and kidney cancer [95,96]. However, studies on FOXP1/2/4 interaction in oncogenesis are lacking. Considering that tissue-specific combination of FOXP2 homodimers and heterodimers may modulate the transcription of specific target genes, it appears crucial to better understand a putative synergy of FOXP2 with its paralogs in oncogenesis.

Proteome-wide surveys report experiments showing the physiological interaction of FOXP2 with 29 other factors (NIH-gene FOXP2 page) [14]. Deciphering which of these partners, such as FOXP1, CtBP1, MAPK3, GATAD2B, might be involved in oncogenic process along with FOXP2 should improve our understanding of gene networks underlying cancer progression.

### 2 Distribution of *FOXP2* gene expression products

#### 2.1 Central nervous system

Transcriptomic analyses have identified *Foxp2* transcripts in mouse cortical neurons mainly, but also in astrocytes and oligodendrocytes, as well as endothelial cells [97]. *Foxp2* is expressed in the neocortex, the striatum, the amygdala, the thalamus, the hypothalamus, the hippocampus and the cerebellum [15,16,98,99]. In the developing human hindbrain, FOXP2 protein has been also detected at strong levels within brainstem nuclei and spinal cord [100]. Brain *FOXP2* expression broadly covers territories responsible for language acquisition and production, including speech-associated motor control, in particular in the developing basal ganglia. The functional consequence of this expression is discussed in the section “4.1. *FOXP2* and language”.

*FOXP2* expression in human brain is elevated during mid-gestation [100], declining postnatally to nearly undetectable levels in adult, while in rodent and zebra finch brain *Foxp2* remains at high levels from neurogenesis through adulthood (Human Brain Transcriptome database) [101]. According to the Human Protein Atlas, female-specific tissues carry more expression than male ones, while in mice Foxp2 protein is significantly higher in multiple regions of the developing male brain compared with females [102].

#### 2.2 Outside CNS in tissues concerned with tumorigenesis

On a system-wide level, besides the brain, *FOXP2* is mainly detected in endocrine (thymus), muscular, cardiac, vascular, pulpmonary, gastrointestinal and urogenital tissues [66,84]. *FOXP2* displays expression in 53 human tissues surveyed - the highest levels being detected in gastro-intestinal and urogenital systems (see UCSC Gencode GTEx, J. Kent and B. Raney).

### 3 Functional properties of FOXP2 in normal conditions

#### 3.1 Subcellular localization of FOXP2

Immuno-colocalization and functional assays have identified a predominant distribution for FOXP2 within the nuclear compartment of the cell. Mutated forms, including the R553H and R328X clinical alleles, have been reported to segregate FOXP2 in the cytoplasm and to preclude their transcriptional activity, due to DNA-binding failure [49]. This misallocation has been hypothesized to induce cellular stress as endoplasmic reticulum stress markers were observed to accumulate in these conditions [103]. As previously indicated, two nuclear localization signals enforce FOXP2 nuclear translocation, with SUMOylation further segregating it in the active heterochromatin. Noticeably, a rare natural isoform of Foxp2 without Forkhead domain has been reported in the mitochondrial compartment of murine cerebellar Purkinje cells [49].

#### 3.2 FOXP2 dimerization

FOXP2 interacts with its paralogs FOXP1/3/4 through heterotypic dimerization, an important feature of its transcriptional activity. Additionally, numerous data have shown that FOXP2 functions may further depend upon interactions with a large repertoire of proteins which may have oncological consequences since some are known to behave as oncogenic drivers in cancer (see section 1.3.2). Among these, complexes including FOXP2 with nuclear receptors might be promising therapeutic candidates. For instance estrogen and androgen receptors colocalize with Foxp2 in mouse amygdala [104], and rat brain [105]. Whether this co-localization translates into real complexes remains unclear.

#### 3.3 Transcriptional activity upon validated FOXP2 target genes

##### 3.3.1 FOXP2 target genes

Differential expression studies across species, including human, non-human primates and animal models, have uncovered vast arrays of FOXP2 transcriptional targets, with bona fide binding sites and functional validation.

In humans two comprehensive studies on FOXP2 target genes have detected 175 and 144 targets in the developing basal ganglia (BG) and in the inferior frontal cortex, respectively (with a 24% overlap); 192 targets in lung tissue (with a 47% and 37% overlap with BG and inferior frontal cortex, respectively) [75]; and 303 targets in human neuron-like cells (with a 14-19% overlap with BG and inferior cortex) [78]. These studies support an involvement of FOXP2 in a diversity of regulatory networks, and some of them may be time and tissue-specific.

To date, most of the studies on FOXP2 targets have been carried out in neural cells or brain tissue, focusing upon the role of FOXP2 in language. There are also some studies on lung [75] and kidney cells [80,106]. Merging all putative FOXP2 targets reported in different tissues, cellular models and mutant models in several species at different developmental stages, we collated more than 1,000 direct or indirect targets genes reported from genome-wide expression studies [17,75,77,78,81,82], as well as individual gene studies [80,106,108-111]. The core analysis for the list of this set of merged targets using Ingenuity Pathway Analysis software (IPA 2017, Qiagen), provided some significant results - in particular, cancer appeared as the top disease. Among the five predominant canonical pathways we noticed the following two:

- VDR/RXR which regulates calcium/phosphorus metabolism and parathyroid hormone secretion, and is involved in immune function and tumor suppression (IPA reports).

- Wnt/b-catenin pathway which is involved in body patterning during development, cellular proliferation, differentiation and apoptosis, and is tightly associated with several cancer entities [112].

Additionally, IPA analysis reported targets of FOXP2 participating in other regulatory pathways including: inflammation, MAPK, Notch, Retinoic acid, Insulin-like growth factor, STAT3, PIK3K/Akt, CREB and TP53. Most of these results are in agreement with previous references [63,75,78]. FOXP2 has also been reported to interact with the Sonic Hedgehog (SHH) pathway [108].

Several studies have described FOXP2 displaying a dual functionality: mostly acting to repress expression, but also activating certain genes. The next paragraphs survey target genes reported to be repressed or activated by *FOXP2*, and collate only candidates individually validated by means of qRT-PCR or *in situ* hybridization.

##### 3.3.2 Transcriptional repressive activity

FOXP2 has been reported to act mainly as a repressor [75,78]. Indeed, the zinc-finger domain of FOXP2 confers transcriptional repressive properties and additionally, FOXP2 interacts with co-repressors, such as CtBP-1 [66,113]. It has been speculated that the repressive activity of FOXP2 might require integration in a multiproteic complex with members from its own family and/or from others such as CtBP-1 [114].

A list of individually validated targets directly or indirectly repressed by FOXP2 is summarized in Table 1. In these studies, genes were selected to investigate the role of FOXP2 in language and neurodevelopment and were mostly assessed in neuronal cell models. We analyzed these genes with Ingenuity Pathway Analysis software: thirty-three of them were involved in tumorigenesis of carcinoma, with high levels of *ASCL1* and *MET* in neuroblastoma formation [115-117], and *PTCH1* in medulloblastoma initiation [118]. Moreover, these genes were also involved in the oncogenic progression of primitive neuroectodermal tumor in IPA analysis. Another gene involved in multiple cancers, *CD164,* has been reported to promote glioma via the tumor-suppressor PTEN [119]. These data suggest that FOXP2 may have the capacity to repress both pro-oncogenic and tumor suppressor genes. Thus, a putative FOXP2-mediated derepression of targets in oncogenic conditions may be complex, occurring only in some phases of the malignancy development, or be tissue/target specific.

**Table 1:** Individually validated targets directly or indirectly regulated by FOXP2 Similar colour code was used for a same reference showing both activated and repressed targets by FOXP2. In bold, targets involved in oncogenesis according to IPA analysis. Such a wide variety of targets involved in cancer suggests that dysregulated FOXP2 may cause complex interaction-dependent effects during oncogenic progression.

	Species	Gene	Model	Technique	References
REPRESSED BY FOXP2	Human	***SLC17A3**, **CALCRL**, **LNPEP**, **HSPB7**, COX11, PM5, **PSEN2**, CD164, **RCN2***	Human neurons (SH-SY5Y cells) transfected with FOXP2 (pcDNA3.1/FOXP2) or EMPTY vector in stable culture.	qRT-PCR EMSA validation	(Vernes et al., 2007)
		***NOS1**, **LBR**, **KCNJ15**, **ANK1***	SH-SY5Y cells transfected with empty vector or FOXP2 isoform I.	qRT-PCR Binding analysis, ChIP	(Spiteri et al., 2007)
		***CNTNAP2***	SH-SY5Y cultured cell expressing different FOXP2 levels.	qRT-PCR	(Vernes et al., 2008)
		***MET*** mRNA and protein level	Overexpressed *FOXP2* in undifferentiated NHNP Cells. MET expression examined 4 d later.	qRT-PCR / WB	(Mukamel et al., 2011)
		*RORg, RXRa, **TNR**, DGAT1, **SLIT1**, BATF3, **AQP1**, **ASCL1**, **CNTNAP2**, **DLL3***	Stable SHSY5Y cells expressing human FOXP2 or the empty vector (EMPTY).	qRT-PCR	(Devanna et al., 2014)
		***CER1**, **SFRP4**, **WISP2**, **SNW1**, **EFNB3***, and ***SLIT1*** in the presence of FOXP2 homodimers	FOXP1/2/4 stably transfected into Embryonic kidney cell line HEK293 cells.	qRT-PCR	(Sin et al., 2015)
		***SRPX2**, uPAR*	Embryonic HEK293 cell line transfected with FOXP2.	qRT-PCR	(Roll et al., 2010)
		***DISC1*** promoter activity and protein expression	Embryonic kidney cell line HEK293 transfected with FOXP2.	Luciferase assay / WB	(Walker et al., 2012)
	Mouse	***Acvr2a**, **Efnb2**, **Wasf1**, **Foxn2**, **Nfat5**, **Nptn**, Nrn1*	Neuroblastoma (neuro2a) cells *in vitro*.	qRT-PCR	(Vernes et al., 2011)
		*Evf1/2, **Nell2**, Nrn1, **Cck**, **Lmo4***	Different brain areas in homozygous mice not expressing Foxp2 protein (Foxp2-S321X) compared with WT.	ISH	(Vernes et al., 2011)
		***Ptch1*** mRNA and protein level (Sonic Hedgehog pathway)	P19 cells transfected	qRT-PCR / WB	(Chiu et al., 2014)
	Rat	**SRPX2** protein level	Dissociated cortical neurons electroporated with FoxP2	WB	(Sia et al., 2013)
ACTIVATED BY FOXP2	Human	*MAPK8IP, SYK*	Human neurons (SH-SY5Y cells) transfected with FOXP2 (pcDNA3.1/FOXP2) or EMPTY vector in stable culture (n.s. in transient culture).	qRT-PCR	(Vernes et al., 2007)
		***CALCRL***	SH-SY5Y cells transfected with empty vector or FOXP2 isoform I	qRT-PCR	(Spiteri et al., 2007)
		*RARb-001, RARb-002, RARb-005-201, RORb, CRABP II, RGS2, SPOCK1, FGF1, ID2, TJP2, NEDD9, SYK, BCL2, ETV1, HES1, NAV2*	Stable SHSY5Y cells expressing human FOXP2 or the empty vector (EMPTY)	qRT-PCR	(Devanna et al., 2014)
		*PRICKLE1, NCOR2, NEUROD2* and *PAX3* in the presence of FOXP2 homodimers	FOXP1/2/4 stably transfected into Embryonic kidney cell line HEK293 cells	qRT-PCR	(Sin et al., 2015)
	Mouse	*let7-d, mir 9, mir216*	Neuroblastoma (neuro2a) cells *in vitro*.	qRT-PCR	(Vernes et al., 2011)
		*PDGFRa*	CTX progenitor cells isolated from the forebrain of mouse embryos electroporated with US2-Foxp2.	IHC	(Chiu et al., 2014)

##### 3.3.3 Transcriptional activating activity

FOXP2 has also been reported to activate expression for some genes in several experiments. However, since these experiments were carried out upon FOXP2 overexpression in culture conditions, it cannot be ruled out that the observed activation of targets resides in the fact that other members of the FOXP2 family are competitively displaced by a surplus of FOXP2, which may impede the formation of multiproteic repressor complexes [114]. Transcriptional activation by FOXP2 may also be explained by differential affinity of FOXP2 for the DNA binding site, cofactors interacting with FOXP2 or post-translational modifications of FOXP2 [114].

Analysis of individually validated genes upregulated by FOXP2 (Table 1) using IPA revealed cancer as the top listed disease. Highlighted genes involve *BCL2* and *HES1,* which are involved with proliferation of neuroblastoma cell lines, and *NEUROD2* which plays a role in neurogenesis of carcinoma cell lines. Additionally, *NAV2* induces neurite outgrowth and is highly expressed in neuroblastoma cells, uterine endometrial stromal sarcoma and colorectal cancer [120]. *MAPK8IP* plays an anti-apoptotic role [121] and showed decreased expression level in glioblastoma [122]. The protein kinase *SYK* has been described as tumor suppressor in breast and glial cells [123].

#### 4 Roles of FOXP2 in normal and non-oncogenic pathological conditions

Close to sixty phenotypes have been reported across eight null mutant alleles for mouse *Foxp2* (MGI:2148705). While most anatomic systems have been concerned with morphogenetic alterations, neoplasm has not been reported so far as being associated with *Foxp2* dysregulation in the mouse. This observation is consistent with the reported resistance of rodents to neoplasm in general [124].

However, lack of *Foxp2* entails juvenile development and leads to morphological alterations that impact CNS tissues (neocortex, cerebellum, basal ganglia), sensory organs (eye, ears), functional activities (vocalization, balance) and statuary growth with death occurring by the end of the first month due to unclear factors. The latter may include pulmonary under-development, but not lack of maternal care or feeding difficulties [20,84]. To some extent, these defects bear some relevance to several aspects of abnormalities reported in human heterozygous patients with defective *FOXP2* expression (see section 5.2.3).

In the CNS under normal conditions, the identification of FOXP2 targets has suggested roles in neurodevelopment (neurotrophin signaling, apoptosis, differentiation, and migration) and neurotransmission (synaptic plasticity, neurite outgrowth, axonogenesis and axon guidance) [75,78,82]. Indeed, FOXP2 was involved in different processes such as neurogenesis [94] including production of interneurons [108], detachment of differentiating neurons from the neuroepithelium [94], neuronal differentiation [108,109,125], migration [109,126], neurite outgrowth [17,82,127], synaptogenesis [111] and dendritic spine morphogenesis [111,128].

Hereafter are briefly overviewed non-oncogenic defects observed in human; the cancers associated with *FOXP2* dysregulation will be subsequently detailed in chapter 5.

##### 4.1 FOXP2 and language

A cardinal feature of *FOXP2* hemi-deficiency in human patients pertains to language [19,36,129]. This association is detailed in the OMIM page for *FOXP2* (OMIM 605317; “Speech-language disorder-1”) and it was first described for the widely studied “KE” pedigree, which carries an arginine-to-histidine substitution at R553H in the DNA Forkhead binding domain [19,36]. Both structural and functional defects impacting language were described in approximately fifteen family members. These include orofacial motor control and articulation, comprehension and expression abilities, as well as non-verbal cognitive skills, with major grammatical failure. Additional unrelated cases of people showing language deficits were also linked to heterozygous or hemizygous mutations of *FOXP2* [29,130-133]. While developmental verbal dyspraxia is a fundamental disorder of this syndrome, its etiology remains debated and might be impacting multiple neural pathways [80,134]. Whether *FOXP2* is involved throughout the construction of the neuro-musculo-skeletal apparatus bearing speech production remains debated.

##### 4.2 FOXP2 and autism

We previously described the association of *FOXP2* with language. Another correlation which remains to be functionally elucidated is between *FOXP2* dysfunction and autism spectrum disorder (ASD). Epidemiological studies rank *FOXP2* with a score of 3 on the SFARI autism scale, an intermediate “suggestive evidence” among stronger scores (Syndromic; 1: high confidence; 2: strong candidate) and weaker ones (4: minimal evidence; 5: hypothesized but untested; 6: unsupported). Importantly, its activities place FOXP2 at the center of an interactome hub regulating the expression of a cohort of other autism-linked genes. This set comprises more than 30 genes reported in different brain regions or cellular models in mouse and human, including syndromic genes (e.g. *CNTNAP2, FMR1, Pax6, MEF2C*), “high confidence” (e.g. *TBR1*), “strong candidate” genes (e.g. *FOXP1, MET*) and “suggestive evidence” susceptibility genes (e.g. *Auts2*) [75,77,78,107,109,110,135]. Additionally, large chromosome accidents comprising the *FOXP2* region are functionally linked to autism (see section 5.2.3). Social communication deficits are central to ASD diagnosis, and both language dysfunction and autism may be influenced through downstream regulation by *FOXP2* key target genes that ultimately impact circuit wiring [110]. One such illustrative interaction lies in the direct repression of the neurexin gene *CNTNAP2* by FOXP2 upon binding to a regulatory sequence in intron 1 [81]. This neurexin has been strongly associated with autism [136], and both genes are expressed in the basal ganglia and amygdala - two important territories of the social brain.

##### 4.3 Other neuropathogenetic processes

Converging data indicate that *FOXP2* is important for modulating the plasticity of relevant neural circuits. Indeed, *FOXP2* appears among the twenty-three clinically relevant genes common to ASD, bipolar disorder and schizophrenia [137]. The affected processes remain still largely unknown.

In accordance with a neurogenic role of FOXP2 in neuropathogenetic processes, one polymorphic variant of *FOXP2*, rs2396753, is associated with hallucinations in schizophrenia and correlated with grey matter reduction [138]. In mice, *Foxp2* null mutants displayed a reduced cerebellum [20], suggesting that *Foxp2* is a key regulator in the development of progenitor cell proliferation and differentiation in this territory. Moreover, reduced *Foxp2* dosage impaired motor-skill learning and synaptic plasticity in mice [139]. In fronto-temporal lobar degeneration, patients carrying *FOXP2* polymorphisms affecting verbal fluency showed hypoperfusion in language-associated brain areas including the left inferior frontal gyrus, and putamen [140].

The neurogenic role of human FOXP2 was assessed in developing mouse cortical cells [141]. FOXP2 appeared to control the behaviour and fate of ventricular zone progenitors by modulating their capacity to engage into neuroglial differentiation. FOXP2 may thus act as a neurogenic switch in the embryonic brain. Lack of this switch may adversely impact neurogenesis by allowing cortical progenitors to remain in a proliferative state, a condition favoring neural oncogenesis. Whether this property subsists throughout post-natal life in brain neurogenic niches remains to be determined.

#### 5 Oncogenic consequences of FOXP2 dysregulation

##### 5.1 Repertoire of cancers reported to involve FOXP2 dysregulation

Attempts to assess whether *FOXP2* transcript or protein levels can be of diagnostic relevance have been collated in cancer databases, along with individual cases reports. However, discrepancies still remain to be solved before a clear understanding of these conditions can be reached. Beyond inter-individual variability, these differences may suggest alternative and tissue-specific roles as tumor suppressor or as oncogene, depending on activated signaling pathways. Furthermore, whether the observed expression levels are causative or consequential to the oncogenic condition has not been systematically assessed.

**Figure 4:**
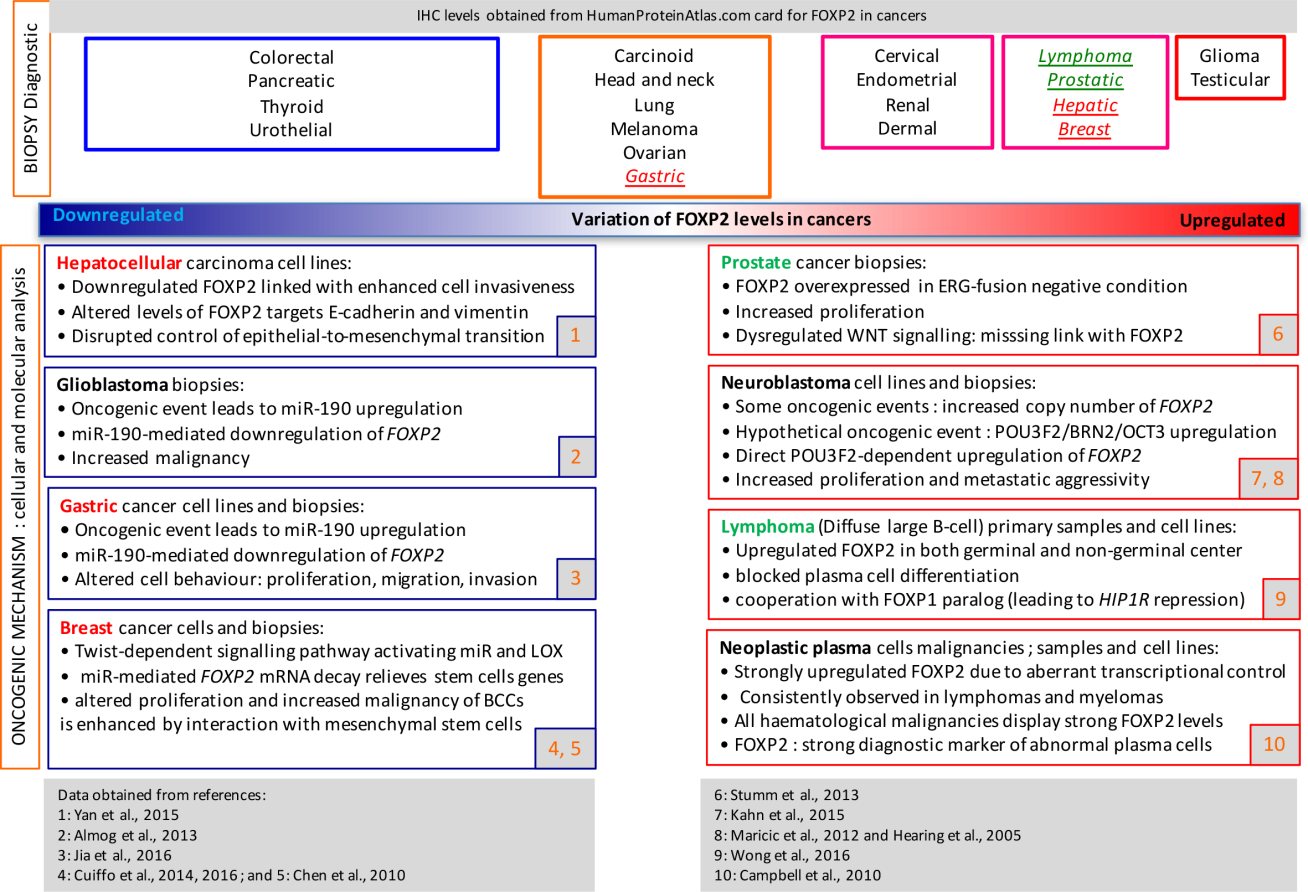
Onco-diagnostic relevance of FOXP2 expression level, comparing immunophenotyping in cancer biopsies (top pannel) with mechanistic data (bottom pannel) Compared regulations: red writing= different results; green writing= identical results.

The ProteinAtlasDatabase displays FOXP2 immunodetection within twenty surveyed cancer types. We categorized these cancer conditions according to FOXP2 levels, relative to healthy tissues (Figure 4, upper panel). While six cancer types displayed unchanged levels, eight appeared moderately increased, and two strongly elevated (glioma and testicular). On the other hand of the spectrum, four conditions displayed moderately reduced FOXP2 immunosignal intensity.

In contrast, in another set of peer-reviewed studies summarized in Figure 4 (lower panel), down-regulated *FOXP2* expression was found in breast cancer (RNA: [9]), hepatocellular carcinoma (protein: [8]) and gastric cancer (protein: [10]). Aberrant levels of FOXP2 factor were found in different types of prostate cancers, strong levels being linked to poor prognosis in ERG fusion-negative prostate cancers [13]. On the other hand, overexpressed *FOXP2* was reported in multiple myeloma, MGUS (Monoclonal Gammopathy of Unknown Significance) and in several subtypes of lymphoma (both RNA and protein [5] or just in protein [11]) as well as in neuroblastoma (RNA and protein [12]).

While the ProteinAtlasDatabase indicates that the overall level of FOXP2 factor in twenty selected cancer conditions may vary in both directions, it thus cannot be ruled out that they reflect secondary impact of oncogenesis on *FOXP2* transcription and/or translation. Genomic information should be further analyzed to complete this survey.

In the following sections (“entities”) we focus upon cancer conditions associated with *FOXP2* dysregulation which are supported by research articles (summarized in Figure 4, lower panel).

###### 5.1.1 Entity: Breast cancer cells

Recent observations have raised a putative tumor-suppressor role for *FOXP2* [9,142]. Whether this property applies to tissues different than the breast mesenchyme remains to be demonstrated. Mechanistically, *FOXP2* translation appears to be actively impaired by two successive waves of dysregulated micro-RNAs: initial MSCs (mesenchymal stem cells)-induced expression of a cluster of microRNAs (miR-199a-214, miR-762) led the activation of a secondary network of microRNAs (miR-1915, *let-7b, and* miR-34a) which subsequently repressed the expression of *FOXP2* [9].

These miRs are encoded by loci which are either intragenic (miR-1915 within *CASC10* intron) or extragenic (let7b; miR-34A; miR-762 colocalized with *BCL7C* intron on the reverse strand; miR-199A2 and miR-214 are both colocalized with *DNM3OS* and miR-199A1 on reverse strand from *DNM2*). Among these genes, the *CASC10* is associated with cancer susceptibility, *BCL7C* is a tumor suppressor and DNM2/3 are P53 activators.

The activation of the TWIST-1 transcription factor has been reported to be responsible in this cascade [9], which remains to be fully detailed. This mechanism seems to concern only breast mesenchymal stem cells, as no cancer cell population has been reported to display reduced *FOXP2* transcript levels in this survey [142]. The chain of ensuing events involves the homing of these cells into the stroma of the initial breast tumor, where they mingle with quiescent cancer stem cells. This interaction potentiates the oncogenicity of cancer stem cells, which triggers a powerful growth and metastasis of the tumor.

Thus, it may be that the tumor suppressor role ascribed to FOXP2 may in fact be indirect, and rather lie within its capacity to normally prevent mesenchymal stem cells from homing into the tumor and/or subsequently activating resident cancer stem cells.

###### 5.1.2 Entity: Hepatocellular carcinoma (HCC)

The Human Protein Atlas suggests a moderate or weak expression level of FOXP2 in sections from hepatocellular carcinoma biopsies. Consistently, a study proposed that reduced FOXP2 protein levels in biopsies might be associated with poor outcome [8]. In this study, established cancer cell lines were used as models to propose that such a low FOXP2 level was causative to their invasive capacity. Whether this assay reflects *in vivo* oncogenic processes in HCC remains to be determined as endogenous hepatic cell FOXP2 contents are already low when compared to other tissues. In line with the previously reported oncogenic mechanism (entity “breast cancer”), it might be necessary to determine whether the observed FOXP2 levels concern endogenous HCC cancer stem cells *per se*, or mesenchymal stem cells prone to HCC homing. Noticeably, the oncogenic course might be more complicated as it has been reported in other cancers (see further) that invasiveness was either associated with *FOXP2* up-regulation as in prostate cancer [13] or, conversely, with *FOXP2* down-regulation as reported for breast cancer [9]. An indirect *in vitro* assay in established human cell lines from hepatocellular carcinoma has shown that malignancy potential was associated with reduced levels of *TALIN2* (*TLN2)* [54]. This gene harbors a microRNA, miR-132, which is predicted to target *FOXP2* transcripts [126]. It may thus be speculated that *in situ*, progressive loss of *TLN2*, and the congruent loss of miR-132, might lead to increased levels of FOXP2 as malignancy progresses.

###### 5.1.3 Entity: Multiple myeloma

FOXP2 is not detected in the normal hematopoietic lineage. Yet the discovery of its strong up-regulation in B lymphocytes from patients afflicted with hematological cancers, including multiple myeloma, has supported the proposal to add it to the arsenal of diagnostic markers, with an even better resolution than those previously available [5]. Whether this up-regulation stems from the oncogenic process or triggers it remains to be formally established in human myelomas. In particular its association with its paralog FOXP1, a strong marker of hematological malignancies, warrants further investigation.

###### 5.1.4 Entity: Diffuse large B-cell lymphoma (DLBCL)

Browsing further blood malignancy conditions identifies FOXP2 as a putative marker for DLBCL, in line with previous observations that FOXP1 is a determinant oncogenic driver in these cancer types [11]. While both factors can be co-immunoprecipitated, whether they cooperate and synergize during transformation remains to be determined, since they might prove both to be strong target candidates as can be read in several FOXP2-related patent proposals.

###### 5.1.5 Entity: Prostate cancers

A large scale (10K+ patients) GWAS transcriptomic survey of prostate biopsies has readily associated FOXP2 levels with cancer outcome [13]. Firstly, nuclear FOXP2 expression in epithelial cells, and not stromal cells, is reduced when compared to normal prostate epithelium. Secondly, advanced stage and severe conditions display strong epithelial staining, especially in ERG fusion-negative conditions - while ERG fusion-positive cancers lacked this association. Interestingly, FOXP2 levels were reported to significantly correlate with cancer cells proliferative activity. Thirdly, in the long-term phase of the longitudinal study, high FOXP2 levels were found to correlate with relapse frequency of ERG-negative cancers. Whether FOXP2 might be a good candidate target in these conditions remains to be determined, as its strong expression in the normal prostate epithelium might be associated with a physiologically relevant role, as for its paralogs of the FOXA family. In the meantime, this study clearly identified FOXP2 expression levels as a strongly discriminative and prognostic tool. In ERG fusion-positive prostate cancer (about 50% of cases) the androgen responsive TMPRSS2 gene fuses to the ETS family transcription factor ERG gene, increasing ERG protein expression. This may activate different pathways and hormonal interactions compared with ERG fusion-negative prostate cancer leading to differential interactions with FOXP2 and distinctive tumor progression.

With regards to oncogenesis mechanism, it should be noted that the *FOXP2* transcript is a *bona fide* target of miR-190, a microRNA lying within an intron of, and concomitantly expressed with, the cytoskeleton-associated protein TALIN2 (TLN2) - with both genes being down-regulated in advanced prostate cancer [143]. Furthermore, *TLN2* hosts another microRNA, miR-132, which also targets *FOXP2* transcripts, and has been widely reported as being involved during prostate cancer progression [144].

An encouraging report has identified another *FOXP2*-targeting microRNA, miR-628, as an efficient tool to damper the aggressiveness of prostate epithelial cancer cell lines [145].

###### 5.1.6 Entity: Gastric cancer

Human gastro-intestinal tract cells express FOXP2, essentially in the glandular compartment. Gastric cancer biopsies display mostly reduced FOXP2 immunoreactivity. Established Human cells lines of the gastric cancer lineage have been shown to display reduced *FOXP2* transcript levels when compared to non-cancerous gastric cells [10]. Which factors may account for reduced FOXP2 expression level in gastric cancer remains to be determined. Candidates, among others, include FOXP2-targeting microRNAs, whether alone or in combination. One, miR-190, has been reported to be up-regulated in both gastric cancer biopsies and in one established gastric cancer cell line [10]. As reported in other sections, miR-190 is often found associated with tumorigenic conditions. One assumption is thus that elevated miR-190 might lead to reduced target FOXP2 transcripts, as observed in established cell lines [55]. However, whether this event is oncogenic to or consequential from gastric cancer development, and whether and when other FOXP2-targeting miRs might be involved, remain two major issues to be solved.

###### 5.1.7 Entity: Glioma

FOXP2 is involved in several aspects of central nervous system ontogenesis, including neural differentiation, axonogenesis, dendritic spine growth, synaptogenesis, neuroblast migration and synaptic plasticity (described in section 4.3). A comparative analysis of neurogenic functions exerted by *FOXP2* in human and mouse has unveiled a new, human-specific activity in the developing cerebral cortex progenitor cells: in contrast to its murine orthologue, *FOXP2* exerts pro-neurogenic activities by promoting the differentiation of human neural precursors and preventing or delaying their proliferation [141]. This property may bear oncogenic consequences since *FOXP2* invalidation relieves neural progenitors from a proliferation repressive signal. This may be related to the etiological event observed in glioblastoma and neuroblastoma associated with *FOXP2* dysregulation [10,12,55], as a side-effect to its role in the acquisition of a stronger neurogenic contingent in the human brain.

In another study examining the pro-apoptotic effect of TP53 activation on transformed glioma cells, we noticed the downregulation of *FOXP2* during cell death of these cancer cells [146,147]. Further studies are required to determine whether such a tumor-suppressing role, under the control of TP53, bears some relevance with *FOXP2 in vivo* function.

###### 5.1.8 Entity: Colorectal cancer

We surveyed longitudinal reports for oncogenic conditions involving FOXP2 deficiency, either repressed or overexpressed, to assess whether it could be considered as a pro-oncogenic player. Although such reports remain scarce, a paper might hold relevant cues [148]: in this paper authors have examined a colorectal adenoma which progressed into a carcinoma condition, by assembling the transcriptomic signature throughout the transition. Among relevant genes, *FOXP2* appeared to be the ninth-most increased among 305 up-regulated genes in precancerous tissue (adenoma), suggesting that it may belong to a group of early genes whose strong expression level was associated with a poor prognosis of tumorigenic progression. We detailed the evolution of the transcriptome throughout this process in section 5.3.2 to illustrate how *FOXP2* dysregulation may associate with large sets of putative FOXP2 target genes during oncogenesis.

Collectively, this work suggests that *FOXP2* in this cancer might belong to a small group of pro-oncogenes associated with “priming” the epithelium for cancer progression. Whether this observation stands true for other conditions, it remains to be elucidated by further large-scale expression signatures in pre- or early malignancy conditions. This knowledge may contribute to improve our understanding of oncogenesis and provide candidate genes for diagnosis and prognosis in biopsies, along with putative therapeutic targets.

###### 5.1.9 Entity: Osteosarcome

In the mouse embryo, *Foxp2* is regionally expressed in the healthy bone by proliferating chondrocytes, collar bone periostal cells but not mature osteoblasts [149]. Consistently, the human osteoblasts lacked *FOXP2* expression. Functional assays on established cell lines from the bone linage suggested that at least *in vitro*, growth arrest resulted from strong FOXP2 upregulation through indirect induction of the cell-cycle inhibitor *p21^CIP/WAF1^*, a p53 target. It is difficult at this point to connect these *in vitro* data with the mechanisms underlying osteosarcoma formation in human [150].

###### 5.1.10 Entity: Other oncogenic conditions

This review focuses on published reports of *FOXP2* involvement at various stages of oncogenic processes in human tissues. Several databases display *FOXP2* expression levels in other cancer types, which we elected to mention, because no associated articles were available at the time of preparation of this review. They include testicular cancer and renal cancer (e.g. Figure 4, upper panel).

##### 5.2 Genome-wide and clinical oncogenomic research involving the FOXP2 locus

Oncogenic conditions have been analyzed with regards to a genomic involvement of either the *FOXP2* locus or regulatory elements controlling *FOXP2* transcription:

###### 5.2.1 Molecular conditions preserving from oncogenesis

Transcriptomic profiling of tumor cells has led to the discovery that cancer stem cells which display a severe malignant and highly metastatic phenotype expressed reduced *FOXP2* levels with regards to normal cells. Functional validation has led to the notion that *FOXP2* repression, mediated by microRNAs, was causal to this phenotype and not consequential, at least in breast cancer [9]. As discussed in paragraphs dedicated to breast cancer and colorectal entities, these observations have raised the possibility that *FOXP2* might actively be part of a protective network of factors involved in preventing pro-oncogenic processes - at least in some tissues.

###### 5.2.2 Molecular conditions associated with oncogenesis

####### 5.2.2.1 The 7:114,629,945 cancer mutation

To determine whether variations in the genomic sequence spanning the *FOXP2* locus were associated with tumorigenesis, we combined data from Ensembl and the Cosmic catalogue (release 81). We extracted phenotypic descriptions associated with 24,804 annotated variants (among 3M+ detected), including 1,155 associated with pathogenic conditions rated “significant”.

**Figure 5:**
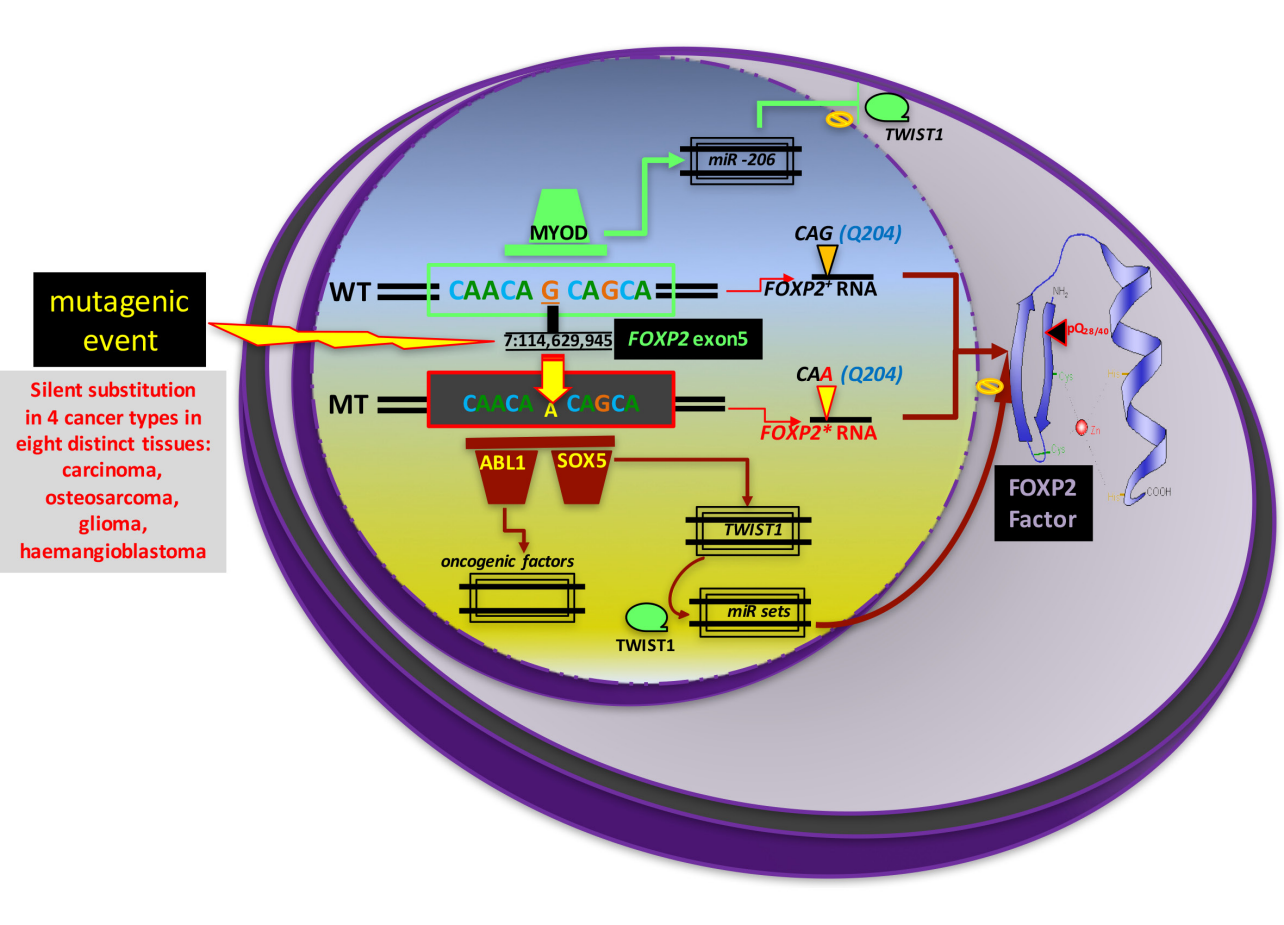
PROTEIN Hypothetic scenarios of oncogenic events involving a known regulatory element embedded in the fifth exon of FOXP2 encoding the polyQ40 stretch. This proposal stems from the observation that six different cancer types, with downregulated *FOXP2* expression, share an identical point mutation at 7:114,629,945 (Q204) in the fifth exon of *FOXP2*. This scheme explores a few of the putative functional consequences of this mutation, considering the observation that this position belongs to a validated promoter. On the one hand the mutation may hinder the fixation of an important transcription factor to this promoter. MYOD appears compatible with this site. On the other hand, this mutation may lead to the creation of a new binding site consensus for factors which normally do not bind this promoter. We represent here two compatible candidates: SOX5 (5′-TWWCAAAG-3′), and ABL1 (5’- AA/CAACAAA/C -3’). Binding of these two factors may have long-range consequences, including for instance the activation of *TWIST1* by SOX5. Transcriptomic data suggest this latter scenario may prove true at least for the breast cancer [9,178].

On the other hand, browsing the Cosmic catalogue returned 531 tumor biopsies displaying a confirmed somatic mutation within the FOXP2 locus. Among those, the top ten most frequent mutations appeared in at least four histological types of cancers (carcinoma, glioma, haemangioblastoma and osteosarcoma) involving eight different tissues (bone, brain, bladder, GI tract, urinary, lung, prostate, thyroid) throughout the survey, respectively up to 22, 19 and 19 times for the three most frequent variants detected at position 7:114,629,945. Interestingly, these mutations (all being G>A substitutions) are silent, as they preserve the Q (aminoacid 204) normally encoded within the exon 5. In Figure 5 we propose a few functional consequences of this mutation.

**Figure 6:**
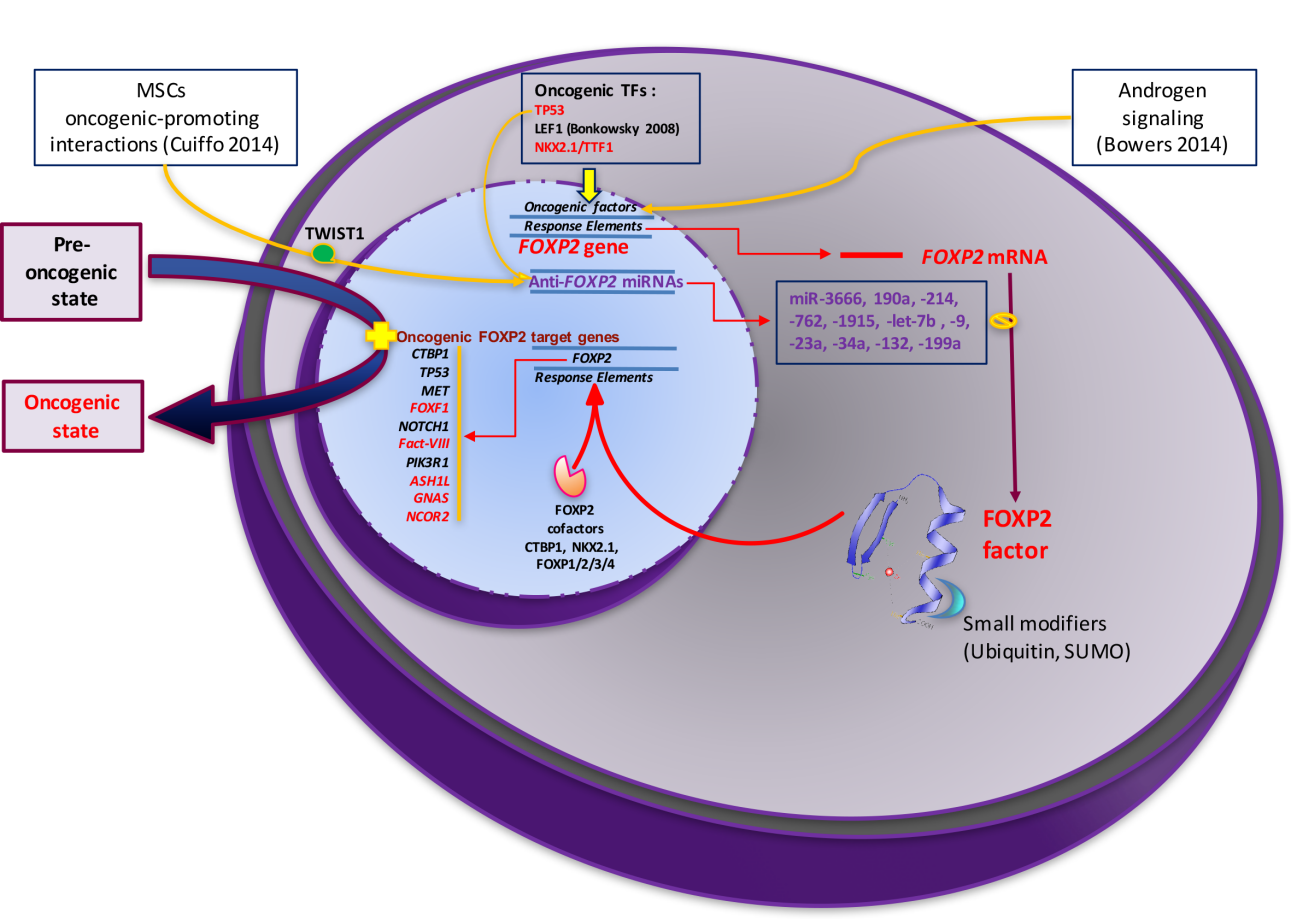
A putative FOXP2-dependent pro-oncogenic/tumor suppressor regulatory network This scheme illustrates how diverse activation pathways may converge to convert a typical cell from a pre-oncogenic to an oncogenic state through abnormal FOXP2 expression and activity. Genes and factors indicated here have been observed in numerous but distinct cancer types detailed in the main text and should not be considered as collectively acting throughout all steps of the oncogenic progression. The illustration of the FOXP2 structure is from Wikipedia.

Importantly, these substitutions neither create nor eliminate donor or acceptor consensus splice sites in the region. We note that this G (114,629,945) is also the first nucleotide of the *FOXP2-219* isoform, a 309nt processed transcript of unknown function (ENST00000634372.1). The pathogenicity of these mutations may thus more probably lie in the chromosomal importance of this 7:114,629,945 nucleotide, and not with its subsequent codon translation.

To address this issue, we examined the genomic context of this position. Using Genomatix software (3.9) we identified putative binding sites which belong to an experimentally validated promoter - located at position [114,628,854..114,630,045], Genomatix Promoter ID GXP_6756854 on the plus strand. This promoter controls the transcription of *FOXP2* transcript short isoforms *FOXP2-225* and *-219*. Analyzing consensus transcription factors binding sites in this territory, involving the 7:+114,629,945 ‘G’ position, yields the myoblast determining factor MYOD (CAGC) as the strongest candidate. In other words, these silent G>A substitutions detected in numerous tumors may alter MYOD binding onto this *FOXP2* promoter. At least two oncogenic scenarios might be at play.

Firstly, this mutated promoter may be unable to control the transcription of the neighboring *FOXP2-225* and *- 219* transcripts. The first is protein-coding, while the second is processed but not translated. In this scenario, one may speculate upon a putative suppressor-like function normally preventing oncogenesis.

Alternatively, this mutated regulatory element may be unable to control longer-distance effector genes, which themselves normally bear anti-oncogenic functions. Whether MYOD exerts any role in either scenario remains to be determined. This issue might be relevant, as MYOD has been reported to exert tumor suppressor functions in numerous tissues involving *FOXP2* mutation: MYOD exerts anti-proliferative actions, and is suspected to play a tumor suppressor role in breast cancer cells [151], as well as in medulloblastoma [152], and in rhabdomyosarcoma of the prostate [153]. We propose that although the *MYOD* gene may not be directly impacted by the oncogenic mutations in these three conditions, the MYOD factor might well be rendered functionally deficient due to its incapacity to bind its mutated target sequence in the GXP_6756854 promoter within the *FOXP2* locus. Thus, this hypothetical oncogenic scenario at play may involve the lack of function of MYOD due to a silent oncogenic mutation within *FOXP2*. Experimental evidence is required to assess this hypothesis. This mechanism involving MYOD may not be in itself of sufficient oncogenic relevance.

Indeed, in parallel, the conversion of this MYOD binding site into one specific for another transcription factor may confer a new binding target for alternative factors. We have found that SOX5 may be a suitable candidate transcription factor accommodating for this new site. Such a conversion may allow for a newly defined transcriptional control of an alternative set of target genes involved in oncogenesis. Here we have illustrated such a possibility with *TWIST1*, a bona fide SOX5 target on the same chromosome as *FOXP2* [154]. TWIST1 may trigger a chain of regulatory events through the activation of miRs; subsequently, this may lead to *FOXP2* downregulation [9].

Another possibility relates to the capacity of the pro-oncogenic factor ABL1 to bind the same sequence. Noticeably, the ABL1 consensus is close to that of HMG-like proteins including LEF-1 and SRY (SOX5). Deciphering the subsequent chain of regulatory events triggered by the binding of ABL1 to this new site would require expensive experimental evidence given the wide array of oncogenic roles exerted by ABL1 [155,156].

Altogether these proposed steps may explain how a single mutation within *FOXP2* may indirectly become a genetic determinant of oncogenesis without affecting *FOXP2* simultaneously in six different types of cancers.

####### 5.2.2.2 Other putative FOXP2 loci of oncogenic interest

A pair of FOXP2 binding regions in the *MET* locus is located at the end of the third intron (in hGRC38 coordinates 7:116,738,696-116,738,727 =caaattaggtactttgagaatcttcccaaatt), which corresponds to the new coordinates of the intronic site reported elsewhere [110]. We found the exact same sequence at 7:116,312,355-116,312,386, which is reported to match HGFR isoforms a/b pre-proteins (synonymous to *MET*), but also falls within the locus of the caveolin *CAV2* gene, further centromeric to *MET* and *FOXP2* on chromosome 7. Dysregulation of this gene has been involved, together with its neighboring paralog *CAV1*, in numerous oncogenic processes [157].

Alternatively, these mutations may slightly impart a consensus binding site (CAGGATAATGA), for the POU6F1 factor, for which oncogenic involvement has been reported [158]. Thus, while not affecting directly FOXP2 factor function, these three most frequently surveyed mutations identify the *FOXP2* locus as a critical region for the prevention of oncogenesis through the action of an intragenic promoter. This may relate to other detected variations within the human lineage for the *FOXP2* sequence, which involve a POU3F2 binding site having a cis-regulatory role [44,159].

In contrast, missense somatic mutations are much less frequent; the first appearing in the list, with three occurrences, being R553W at position 7:114,659,632. Other studies at this position have already identified it as critical for the proper nuclear translocation of FOXP2 - a widely known allele being the R553H variant reported throughout the pedigree of the language deficient “KE” family (described in section 4.1). One may speculate that in skin cells, FOXP2 exerts an anti-proliferative role which is impaired by the mutation - which may be determinant for skin oncogenesis. It remains to be determined whether FOXP2 R553W can translocate into the nucleus of affected skin cells or translocation is dampened as for R553H (see section 1.3.1).

The mutational profile in *FOXP2* within cancers assembled in the Intogen database, displays an accumulation of mutations in the 7:114,270,000-114,270,020 region. Scanning this territory suggests a TAAT box, among others, but no further salient functional motifs. Genomatix analysis of this territory does not identify a validated promoter region, and PROMO.com detection of consensus binding sites identifies a series of putative transcription factors. Among those, TFII, GATA and C/EBP families might appear as the most interesting ones with regards to either transcriptional initiation or oncogenesis, respectively. Further detailed analysis of this territory falls beyond the scope of this review.

Computing cumulative occurrences of mutations across tissue types provides an interesting cue for the oncogenic relevance of *FOXP2*. Using INTOGEN we compared those of *FOXP2* with those of the *TP53* tumor suppressor gene. Most cancer types displayed a 70-90% range of frequencies for *TP53*, which is consistent with its ubiquitous expression and anti-oncogenic role. In contrast, *FOXP2* was associated with an 8-10% score at most in digestive and neural cancers - and averaged a few percent across the rest of the panel. This comparison, while not ruling out a protective role for *FOXP2*, may be indicative of a more restricted role in preventing cancer initiation.

In conclusion, while our analysis highlights variants which have been detected in tumor biopsies, it remains to be formally demonstrated that these are causative oncogenic events.

###### 5.2.3 Large chromosomal accidents not associated with oncogenesis

To survey the involvement of the *FOXP2* locus in oncogenic conditions reported in human patients, we browsed the databases detailed below, using either “*FOXP2*” or “HSA7:[114,063,327 .. 114,693,777]” as query terms. To date (February 2018), in DECIPHER database thirty-two patients have been diagnosed with chromosomal accidents involving large portions of chromosome 7 in the vicinity of the *FOXP2* locus: (i) Four of these hits are punctual and three within the 7:114,086,327-114,693,772bp *FOXP2* locus. One is outside at 114,066,645bp (close upstream to the start of several transcriptional isoforms); and (ii) the seven others span a region from 0.7 to 23.7Mbp located either within, covering part or entirely including this locus and its immediate neighborhood. Large chromosomal accidents spanning the 114.1-114.7Mb of *FOXP2* were specifically: Hsa7: 96.1-119.8; 113.6-126.1, 114.0-114.8, 109.1-115.1, 108.8-117.6 and 112.1-119.2Mb. The phenotypic analysis of these six patients has led to sort them into morphogenetic (craniofacial, appendage) and functional deficiencies (language, speech) as well as social disorders (mainly autism-related conditions). Despite this rather large survey of the *FOXP2* locus region, none of these 32 patients have been reported so far in this database to harbor oncogenic process-related issues.

As an alternative source of oncogenic data related to lesions affecting chromosome 7, we filtered the database Chr7.org [160] for the *FOXP2* locus and the occurrence of neoplastic phenotypes. However, the seven reported cases to the last update (August 18, 2004) appear either spared from, or have not been diagnosed with such malignant process their phenotype being mainly associated with language deficits.

Over the past decade efforts have been produced to reconcile oncogenic knowledge from different databases to gain insight into the malignant processes. For deeper and updated analysis of a putative oncogenic role of *FOXP2*, we refer the reader to the series of gene lists compiled on the Buschman laboratory website.

##### 5.3 Hypothesis for a FOXP2-dependent oncogenic gene regulatory network

###### 5.3.1 Putative underlying FOXP2 interactome in cancer

The molecular mechanisms through which FOXP2 exerts its transcriptional activities in an oncogenic situation remain to be elucidated at the biochemical level.

*In vitro* dissection of FOXP2 dimerization with its paralogs FOXP1 and FOXP4 in human cells has shown that the fate of neuronal-specific targets genes of FOXP2 depends upon the composition of the dimers: FOXP2 associated with FOXP1 does not exert the same activity upon FOXP2 target genes than when it is dimerized with FOXP4 [64]. Whether FOXP2 is coexpressed with - and which - FOXP paralogs in pre-oncogenic cell context remains to be established. Furthermore, transcriptomic analysis of cells undergoing oncogenic transformation needs to be performed to determine whether these FOXP2 target genes are expressed and involved in the oncogenic process. These gaps in our knowledge nevertheless emphasize that the study of an oncogenic role by FOXP2 requires a global understanding of its interactions with other Forkhead factors, targets, as well as other upstream regulators including microRNAs, enhancers and co-factors as CtBP1 (described in section 1.3.1 and 3.3.2), which may modify the transcriptional regulatory activity of FOXP2. A prominent example of regulatory element controlling *FOXP2* transcription is MYOD: binding onto *FOXP2* promoter exerts anti-proliferative actions (section 5.1). Anomalous regulatory activity of FOXP2 due to aberrant upstream regulation as well as to mutations in *FOXP2* locus (or in regulatory elements) (see section 5.2.2) might be key in triggering oncogenesis.

Putative molecular involvement of FOXP2 during cancer initiation, maintenance and metastasis processes may relate to FOXP2 ability to differentially modify the expression of target genes linked to several signaling pathways (see section 2.3.1). Screening canonical molecular pathways for putative targets of FOXP2 we observe that from angiogenesis/neovasculogenesis to glucose metabolism and apoptosis, different cancer-promoting physiological processes might be impacted by FOXP2 dysregulation.

IPA analysis using only targets individually studied (collated in Table 1) suggests involvement of FOXP2 in several pathways dysregulated in oncogenic processes, including: NOTCH1, inflammatory response, Wnt/b-catenin, STAT3 and P53.

FOXP2 interactome includes genes from the canonical NOTCH signaling (e.g. *PSEN2*, *DLL3*, *HES1*) and Wnt/b-catenin signaling pathway (*SFRP4*, *ACVR2A*). All of these genes have been previously related to cancer conditions (IPA reports). Additionally, *LEF1* and *MET,* tightly related to the Wnt/b-catenin signaling pathway [161], are strongly associated to FOXP2 (for *LEF1* see section 1.1.3.2) (for *MET* see sections 1.1.1, 1.1.2.2, 5.2.2 and 5.3.3), and both of them have been proposed as biomarkers for prognosis and targets for cancer treatment [41] [24].

Tumor development requires vascularization of the area for the supply of nutrients, primarily glucose. FOXP2 has been reported to upregulate *BCL2* [109], involved in the VEGF pathway (mediating vascularization), as well as in glucose metabolism and glucocorticoid receptors, in tumor suppressor P53 pathway and inflammatory response.

Additionally, FOXP2 interacts with genes modulating the inflammatory response through cytokines, chemokines and angiogenic factors. In cancer and epithelial cells exposed to carcinogens, cell survival and proliferation are regulated by such inflammatory response as well as from apoptotic pathways involving targets of FOXP2 including: *Cyclin D*, *c-MYC* [9] and *BCL2* [179]. IPA analysis provided additional FOXP2 targets participating in inflammatory processes (e.g. *NFAT5*, *SYK*, *PSEN2*, *ACVR2A*) as well as in glucose metabolism and glucocorticoid receptors (*NFAT5*, *NCOR2*). According to IPA analysis, several FOXP2 targets involved in oncogenesis were associated to different pathways, including: ERK/MAPK signaling (*HSPB7*); dopamine (*NOS1*, *KCNJ15*), SHH pathway *(PTCH1)* [108].

During tumor progression, dysregulation of pathways involved in embryonic development is commonly observed — mainly those associated with cell proliferation. SHH pathway is involved in cell differentiation, proliferation and tissue polarity, and is found to be hyperactivated in many solid tumors. Ectopic expression of SHH is sufficient to induce basal cell carcinoma in mice [26]. Among other possible associations with developmental genes, *FOXP2* was reported to downregulate *PTCH1* mRNA and protein levels involved in SHH pathway [108].

Further functional links between FOXP2 and cancer through dysregulation of other signaling processes may warrant examination. Among those we speculate that estrogen/androgen pathways relate FOXP2 and oncogenesis [104,105] (see section 5.1.5. Prostate cancer). Estrogen/androgen pathways are crucial in glandular cancers including breast or prostate cancer. Among others, estrogen receptor signaling comprises several oncogenes targets of FOXP2, including CDK8 [82,159]. Noticeably, the FOXP2 binding partner and paralog FOXP1 was found upregulated and directly activated by estrogen signaling in both breast cancer cells and biopsies [162].

In summary, FOXP2 activity may be in a hub of different pathways which are important oncogenic contributors. The involvement of FOXP2 to activate one pathway or another in oncogenesis may depend upon its interaction with paralogs, cofactors and additional regulatory elements that require further investigation.

###### 5.3.2 Dysregulated FOXP2-dependent genes in cancers

The putative involvement of FOXP2 in so many different oncogenic processes may result from an intrinsic property of this factor to differentially regulate, or cooperate with general oncogenesis-associated genes, including both proto-oncogenes and tumor suppressor genes. For the most widely reported of those genes, we examined the possibility that FOXP2 might control their transcription upon binding in their regulatory elements.

The *C-MET* proto-oncogene has been involved in numerous oncogenic conditions [163], including those for which we examined the contribution of *FOXP2*. Different regulatory networks may be intervening. In particular, *C-MET* expression is regulated by the p53 tumor-suppressor [24]. Additionally, *MET* transcription falls under the control of the AP1-NFAT complex [110,164]. AP1 binds the *MET* promoter at its 7:116,671,439 target binding site (cagaAATTtgagttattatagta). In contrast, FOXP2 prevents *MET* expression through competitive binding with AP1, at the same position (underlined in the above sequence) [110], and crystallisation of the ternary complex DNA/(NFAT-AP-1)/FOXP2 has shown that FOXP2 further impairs AP1 transcriptional competency [165,166]. Altogether, these data suggest that a dual oncogenic mechanism may be at play, whereby normal cellular homeostasis and oncogenesis protection exerted by MET in normal conditions might be lost, due to the competitive displacement of AP1 by FOXP2 from the *MET* promoter. This scenario relates strongly to the oncogenic mechanism reported in immune cancers involving FOXP3-mediated displacement of AP1-NFAT from an interleukin gene [167].

Transcriptional regulatory activity of FOXP2 may be modified by the co-factor CtBP1 (described in section 1.3.1 and 3.3.2) and this interaction may have important oncogenic consequences considering the tumor-promoting activity of CtBPs. CtBPs stimulate epithelial-mesenchymal transition, tumor cell migration and invasion, inhibit apoptosis and repress several tumor-suppressor genes. Conversely several tumor-suppressors target and downregulate CtBPs [67].

We also explored the interaction between FOXP2 and the genes involved in the progression of cancer. The differential expression of genes along the transition from normal to dysplastic epithelium and then to carcinoma has been assessed for colorectal cancer [148]. We exploited these data to draft a putative network of FOXP2-dependent target genes modified before or during cancer progression. First we assembled a database of known FOXP2-associated genes (see section 3.3.1), and crossed it with a set of genes modified by at least two-fold, along the progression of colorectal cancer (CRC) malignancy [148]. However, since this last list [148] contains multiple entries for the same genes, without specifying their raw fold-change values, it is difficult to obtain a clear view of the oncogenic evolution of the transcriptome. This appears especially important as the values reported on the histograms do not reflect the position of the candidates according to their descending order of fold-change in the table. Overall, this lack of clarity makes the task to design a CRC stage-specific signature challenging. Interestingly, four candidates have been validated by immunocytochemistry on stage-specific biopsies, which may be helpful to start defining a diagnosis strategy. This has been performed in another study where the two most downregulated candidates, *DEFA5* and *DEFA6*, have been independently assessed as good markers of colon cancer progression [168]. In the above mentioned CRC study, FOXP2 was highly upregulated upon transition to cancer stage [148]. This was simultaneous with downregulation of some of its target genes including: *HOXB5*, *PLA2R1* and *NPTX2*. *HOXB5* has been reported to be over-expressed in various cancers and knocking it down inhibited metastasis [169,170]. In contrast *PLA2R1* is considered a repressed tumor-suppresssor in several oncogenic conditions [171]. Whether the downregulation of these three FOXP2 target genes is consequential to an increase in upregulated FOXP2 activity remains unclear. In contrast, we found a set of FOXP2-associated genes which were upregulated during successive steps of cancer progression. Based upon IPA assignment, these genes are involved in oncogenic processes and stages such as proliferation, primary cancer establishment, invasion and metastatic tumor: *CALD1, CYR61, EPHA2, PLAUR, THBS1, ZFP36* among others.

Additionally, using Genecard we inspected the promoters of widely reported tumor suppressor genes for the presence of *FOXP2* consensus binding sites. We found them in a wide number of tumor suppressor genes including: *TP53, APC, RB1, VHL, BRCA1, BRCA2*. These putative FOXP2-dependent genes may be involved in preventing oncogenesis in different tissues. One may speculate that an oncogenic scenario involves the loss of FOXP2-dependent regulation of these oncogenic guardians, which may subsequently favor oncogenesis.

###### 5.3.3 Chemotherapy and drug resistance

We explored the possibility that FOXP2 might also exert a role during a later phase of the oncogenic process, in the active elimination of therapeutic agents by cancer cells. This activity is a hallmark of drug-resistance aggressive cancers such as glioblastoma. It is mediated by transporters which expel the chemical compounds, and belong to the ABC family of transmembrane shuttling proteins [172]. ABCA6 and ABCG2 showed aberrant expression in different types of cancers [173,174] and are direct FOXP2 target genes [78,82]. One oncogenic promoting scenario would be the down-regulation of *FOXP2* during the initial phases of tumorigenesis, paving the way for the de-repression of the transporters. Other genes related to FOXP2 that may be involved in cancer drug resistance by drug efflux according to IPA analysis of FOXP2 targets include: *FOXO1*, *RRAS*, *MAPK3*, *PIK3R1*, *MRAS* and *PIK3CB*.

## CONCLUSIVE REMARKS: A DUAL ROLE FOR FOXP2?

Throughout this review we have focused upon published reports involving *FOXP2* dysregulation during cancer progression. We have taken care to separate this process according to tissue type. *FOXP2* is not as ubiquitous as the tumor-suppressor *TP53*, but its involvement in such a wide diversity of tissues may underlie a more general property of this gene. Indeed, phenotypes associated with loss- or lack-of function of *FOXP2* fall into two large functional categories.

The first includes neurodevelopmental defects leading mostly to dysfunctional speech associated structures, including both intellectual and motor skills required for speech production. The second category covers oncogenic defects resulting from dysregulated *FOXP2* levels. It may stem from its capacity to control cell cycle progression, cellular adhesive properties, and cancer cell aggressiveness. Overall, the central nervous system displays involvement of *FOXP2* in both of these categories. In developing cerebral and cerebellar cortices, the proliferating progenitors are kept under tight neurogenic control by post-mitotic cells. Failure of this proliferation checkpoint may lay adverse conditions conducive to neuro- and gliogenic cancer progression. Simultaneously, dysregulated neurogenesis may impair proper neuro-differentiation as well as timely and functional establishment of neuronal circuits, including those recruited for language and speech production.

Aberrant expression of FOXP2 was detected in diverse cancer types, including up- or down-regulated FOXP2 levels depending on the type of cancer, with few discrepancies between peer-reviewed studies and ProteinAtlasDatabase (Figure 4). This suggests that aberrant FOXP2 levels may play either a pro-oncogenic or a deficient tumor-suppressor role that may be tissue-specific and vary along the progression of cancer. Such a dual role of FOXP2 might be ascribed to differential activation of numerous gene targets of FOXP2 and regulatory networks that could affect oncogenic phenotypes independently. Differential target gene/pathway activation may be a consequence of a complex control of *FOXP2* regulation (including micro-RNAs, different combinations of FOXP1/2/4 dimerization, isoforms, alternative splicing, post-translational SUMOylation, interaction with other transcription factors, promoters and other regulatory elements detailed in sections 1 and 5.3) combined with intrinsic binding properties of FOXP2 to different DNA sequences [65].

In the present review, we have described several mechanisms relating FOXP2 to carcinogenesis. They enlighten that further research on the regulation of FOXP2 and the differential downstream regulatory activity of FOXP2 may be crucial to address both roles of FOXP2 in oncogenesis, as well as in language and other neurological deficits (see section 4). This complex regulation is also supported by the fact that *FOXP2* structure is among the most evolutionarily conserved (only 2 amino-acids differ between human and chimpanzee) [17,175]. Thus, even if conservation also applies for some regulatory elements of FOXP2 that are common in both zebrafish and humans (see section 1.1.3.2), essential differences in control mechanisms and regulatory elements of FOXP2 might explain differential downstream activity and functions of FOXP2 in “speaking” versus “non-speaking” species, as well as in oncogenic *vs.* normal conditions.

It is worth noticing that most of the studies addressing FOXP2 in cancer entities reported aberrant levels of expression without providing a causative oncogenic event. To our knowledge, only one study mechanistically linked *FOXP2* with the acquisition of metastatic traits, evidencing association of down-regulated FOXP2 with malignant breast cancer [9]. Parallel comprehensive studies in other cancer types will help to better understand the roles of FOXP2 in oncogenesis.
